# Efficacy, T cell activation and antibody responses in accelerated *Plasmodium falciparum* sporozoite chemoprophylaxis vaccine regimens

**DOI:** 10.1038/s41541-022-00473-1

**Published:** 2022-05-31

**Authors:** Javier Ibanez, Rolf Fendel, Freia-Raphaella Lorenz, Patricia Granados-Bayon, Sina Brückner, Meral Esen, Mihály Sulyok, Zita Sulyok, Steffen Borrmann, Petra Bacher, Alexander Scheffold, Stephen L. Hoffman, Peter G. Kremsner, Benjamin Mordmüller

**Affiliations:** 1 Institute of Tropical Medicine, University of Tübingen, Tübingen, Germany; 2 German Center for Infectious Diseases (DZIF), Partner Site Tübingen, Tübingen, Germany; 3 Centre de Recherches Médicales de Lambaréné (CERMEL), Lambaréné, Gabon; 4 Institute of Immunology Christian-Albrechts-University of Kiel, Kiel, Germany; 5 Institute of Clinical Molecular Biology, Christian-Albrechts-University of Kiel, Kiel, Germany; 6 Sanaria Inc, Rockville, MD 20850 USA; 7 Department of Medical Microbiology, Radboud University Medical Center, Nijmegen, The Netherlands

**Keywords:** Malaria, Live attenuated vaccines

## Abstract

Repeated direct venous inoculation of *Plasmodium falciparum* sporozoites (PfSPZ) together with antimalarial chemoprophylaxis (PfSPZ–CVac) is the most potent way to induce sterile immunity against *P. falciparum* infection in malaria-naive volunteers. However, established schedules are complex and long. Here, we tested two accelerated three-dose schedules (28- and 10-day regimen) assessing efficacy by controlled human malaria infection (CHMI) against placebo, comparing vaccine-specific T cell and antibody responses by antigen-reactive T cell enrichment (ARTE) and protein microarray, respectively. Both regimens were similarly efficacious (67 and 63% vaccine efficacy) but different in the induction of vaccine-specific T cells and antibodies. The 10-day regimen resulted in higher numbers of antigen-specific CD4+ effector memory pro-inflammatory T cells and a broader antibody response compared with the 28-day regimen. Usually in nature, *P. falciparum* liver stage lasts about 6.5 days. The short vaccination-interval of the 10-day regimen prolongs the time of continuous exposure to liver-stage parasites, which may explain the stronger response. Besides dose and number of vaccinations, duration of liver-stage exposure is a factor to optimize PfSPZ–CVac immunogenicity.

## Introduction

The global burden of malaria declined in the first fifteen years of the XXI century, but since then, it has reached a plateau. The latest world malaria report published in 2021 by the World Health Organization even shows that the malaria cases and deaths increased in the last year to reach 241 million cases and 627 000 malaria deaths^1^.

The COVID-19 pandemic has compromised the goals of the Global Technical Strategy to Combat Malaria 2016–2030^2^ and put the progress made over the past decades at risk. Effective SARS-CoV-2 vaccines were developed within less than a year and have a major impact on the pandemic. Likewise, an effective vaccine against malaria^3^ could be a game changer. Proof-of-concept studies for malaria vaccines in humans were first conducted about 50 years ago using irradiated *Plasmodium falciparum* (Pf)-infected mosquitoes^4,5^. Only very recently, the WHO endorsed the first malaria vaccine, RTS,S, for widespread use^6^. Even though this new vaccine might save thousands of lives per year, it is far from optimal as it protects children in endemic areas only for approximately 30%^7^.

The recent development of methods to manufacture aseptic, purified, cryopreserved Pf sporozoites (PfSPZ) from mosquito salivary glands under GMP conditions for injection into humans has great potential to further improve the malaria vaccine landscape and makes attenuated whole-parasite vaccines a leading candidate in the field^8^.

The most potent immunization strategy known so far uses replication-competent sporozoite (SPZ)-stage parasites that are attenuated in vivo. Here, attenuation is achieved using an antimalarial drug that allows progressing of infection from inoculation to maturation in the liver, the clinically silent phase of the infection, but is highly active against asexual blood-stage parasites (e.g., chloroquine). The concept of in vivo attenuation or “infection-treatment” is uncommon in human medicine, but successfully established in veterinary medicine and widely used in the prevention of East Coast Fever (ECF), a disease of cattle caused by *Theileira parva*. This veterinary vaccine shows high efficacy in preventing clinical ECF and mortality^9^.

A second approach to immunize individuals using attenuated PfSPZ is the so-called PfSPZ Vaccine, in which sporozoites are rendered replication-defective by γ-irradiation. The superior potency of in vivo attenuation over approaches using replication-incompetent PfSPZ is likely due to the prolonged exposure to liver-stage parasites, a higher antigen load, and a broader immune response^10^.

Previously and as part of the present study, we established an immunization regimen dubbed PfSPZ chemoprophylaxis vaccine (PfSPZ–CVac), with three inoculations of cryopreserved PfSPZ (PfSPZ Challenge), given every four weeks to volunteers under chloroquine chemoprophylaxis for 10 weeks^10^. Three direct venous inoculations (DVI) of 5.12 × 10^4^ PfSPZ (on days 0, 28, and 56) protected all volunteers from infection, whereas lower doses dose-dependently led to partial protection^10^.

The immunological mechanism behind PfSPZ-mediated protection is not known. We have previously shown that vaccine efficacy (VE) is associated with a higher number of circulating memory CD4+ T cells producing IFN-γ, IL-2, and/or TNF-α following in vitro stimulation with PfSPZ or infected red blood cells (iRBC)^10^. Antibody titers against Pf circumsporozoite protein (CSP), the main surface protein of PfSPZ, increased with dose, but were not associated with protection^10^. However, the immunization scheme of our previous study was still very complex and lengthy. For the development toward a vaccine with better applicability in travel medicine as well as in endemic settings, the time span of the regimen needs to be significantly shortened. The present study reports the results of two more realistic vaccination regimens. The number of interventions was reduced and the schedule shortened to 28 and subsequently down to 10 days.

Regarding immunogenicity, we characterized humoral responses by ELISA and protein microarrays, as well as cellular immune responses. The antigen-reactive enrichment methodology^11^ (ARTE) using Pf*-*infected red blood cells (iRBC) as proxy of Pf*-*infected hepatocytes was implemented to monitor ex vivo, the CD40L+CD4+ T cell specificity during immunization and before the day of challenge (C-1). ARTE has been successfully employed previously to explore antigen- specific CD4+ T cell subsets generated against various infections and immune pathologies^12–15^ but not yet to characterize the immune response of any malaria vaccine candidate. The two-step technique, based on the magnetic isolation of fresh cells bearing the surface ligand CD40L (CD154) plus the intracellular detection of the same CD40L marker in combination with other pro-inflammatory cytokines such as TNF-α^14^, aimed not only to detect the bona fide T-helper cells, but also allowed to distinguish cognate responses within the naive (CD45RO−) or memory (CD45RO+) T cell repertoire.

The longer PfSPZ–CVac regimen with PfSPZ inoculations on days 0, 14, and 28 using repeat inoculations with a timing that allows three separate liver-stage developments had a similar VE but surprisingly was less immunogenic than the shorter one with PfSPZ inoculations on days 0, 5, and 10. In the shorter vaccination schedule, the booster vaccinations are given when the parasites of the previous dose are still in the liver stage. This results in a total period of 16 days during which parasites are constantly present in the liver.

## Results

### Participants, safety, and reactogenicity

Volunteers were allocated to a placebo group (normal saline, *n* = 6) or to a vaccine group with three direct venous inoculations (DVI) of 51,200 PfSPZ either 14 days (*n* = 10) or 5 days (*n* = 9) apart, for a total immunization regimen of 28 and 10 days, respectively. Basic demographic data are summarized in Supplementary Table 1 and the CONSORT diagram is shown in Supplementary Fig. 1. All volunteers received chemoprophylaxis with chloroquine. In addition, half of the volunteers (*n* = 5 verum, *n* = 2 placebo) of the 28-day regimen also received 2 g of extended-release azithromycin (Zithromax Uno) on the day of the first vaccination. The rationale to use azithromycin was based on studies in mouse models in which the drug allowed the plasmodial liver stage to undergo schizogony, but meanwhile blocked the biogenesis and inheritance of the apicoplast. The azithromycin-treated animals did not develop patent asexual blood-stage parasitemia and azithromycin-mediated attenuation led to pre-erythrocytic immunity comparable to chloroquine prophylaxis^16,17^.

Twenty-five volunteers were enrolled in the study and received at least one immunization (intention-to-treat—ITT), 22 participants completed the trial as per protocol (PP). Two volunteers (1 placebo, 1 PfSPZ–CVac 10-day regimen) withdrew from the study following the third vaccination (98 and 74 days, respectively) and before controlled human malaria infection (CHMI) (Supplementary Fig. 2). In all but one volunteer (PfSPZ–CVac 28-day regimen without azithromycin), chloroquine treatment was fully effective in treating parasitemias in the first asexual blood cycle (Supplementary Fig. 2). The individual that unexpectedly developed the parasitemia (178 parasites/µL) during immunization was excluded from the study. Circulating parasites isolated from this participant were fully sensitive to chloroquine ex vivo (50% inhibitory concentration: 3 nM). Traces of chloroquine were detected in plasma, although concentration was below the level of quantification of the assay (<5 ng/mL). In contrast to this, three other randomly selected volunteers had chloroquine plasma concentrations of 98, 119, and 115 ng/mL, which is well above the inhibitory concentrations. Chloroquine was administered at the clinical trial site in the presence of a physician and a second team member, but swallowing was not monitored (e.g., by oral inspection). It is most likely that the participant who unexpectedly developed the parasitemia did not swallow the tablet but kept it in the oral cavity and discarded it when leaving the examination room. The volunteer was successfully treated with atovaquone-proguanil but disagreed with further pharmacokinetic investigations.

A total of 313 adverse events (AE) were reported in 25 randomized volunteers. No serious AE (SAE) occurred. In total, 194 AEs were reported during the immunization (Supplementary Fig. 3 and Supplementary Table 2) and 119 AEs during the challenge infection were solicited (Supplementary Table 3). The most frequent clinical AEs were headache (20 episodes), fatigue (20), and diarrhea (12). The most frequent laboratory AEs were increased alanine (7) and aspartate (5) aminotransferases (ALT and AST, respectively), elevated lactate dehydrogenase, LDH (8), and high monocyte count (9). In total, 276 AEs were mild (Grade 1), and 27 were moderate (Grade 2). Six severe Grade 3 AEs were reported, including increased ALT, lymphopenia, and thrombocytopenia associated with the case of malaria during immunization. Lymphopenia and two Grade 3 gastrointestinal bleedings were reported in the 10-day group. All Grade 3 AEs resolved uneventfully.

### Parasitemia kinetics during vaccination and vaccine efficacy

**Fig. 1:**
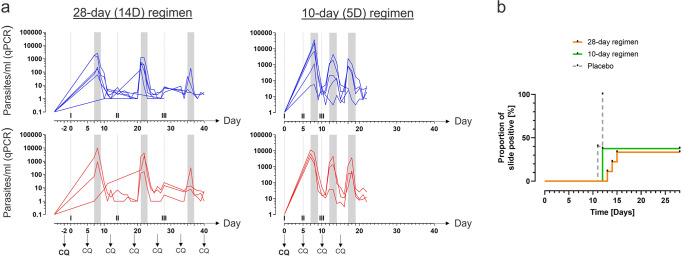
Submicroscopic infected red blood cells (iRBC) are detectable during the vaccination period. **a** The kinetics of the parasites detectable by RT-qPCR in the peripheral blood during the vaccination period using PfSPZ–CVac is shown. The left panels represent the parasitemia during the 28-day regimen and the right panel represents the 10-day regimen. Individuals being protected against the subsequent CHMI are shown in blue, unprotected are shown in red. Gray vertical bars represent the expected 7–9-day prepatent period after direct venous inoculation (DVI). Arrows indicate the time points when participants received chloroquine (CQ) tablets. **b** Proportion of slide positivity after CHMI is depicted using a Kaplan–Meier plot.

Circulating asexual blood-stage parasites were detectable 7–9 days following each vaccination in the 28-day regimen. Peak parasitemia tended to decrease during sequential vaccinations. This trend was more pronounced in volunteers who were later protected against CHMI (Fig. 1a, Table 1). Parasitemia also peaked approximately 8 days after each vaccination in the 10-day regimen with decreasing peak parasitemia after the second and third DVIs. Here, the decrease in peak parasitemia was not associated with protection. All five placebo recipients developed parasitemia following inoculation of PfSPZ Challenge. VE was 67% (6/9, *p* = 0.000045) and 63% (5/8, *p* = 0.012) in subjects immunized at 14- and 5-day intervals, respectively, as we have reported before^10^ (Fig. 1b).

**Table 1:** Kinetic of transient parasitemia during the vaccination period in the 28-day and 14-day regimen in protected and unprotected individuals.

Vaccine dose (PfSPZ)	Vaccination time points	CHMI outcome	Immunization	Median parasitemia (# PCR positive/# total participants)	Day peak parasitemia
51200	0,14,28	Protected	I	204 (5/6)	8 (+8)
51200	0,14,28	Unprotected	I	933 (2/3)	8 (+8)
51200	0,14,28	Protected	II	457 (6/6)	21 (+7)
51200	0,14,28	Unprotected	II	2378 (3/3)	22 (+8)
51200	0,14,28	Protected	III	3 (4/6)	36 (+8)
51200	0,14,28	Unprotected	III	14 (3/3)	36 (+8)
51200	0,5,10	Protected	I	6953 (5/5)	8 (+8)
51200	0,5,10	Unprotected	I	6556 (3/3)	7 (+7)
51200	0,5,10	Protected	II	215 (5/5)	13 (+8)
51200	0,5,10	Unprotected	II	1372 (3/3)	13 (+8)
51200	0,5,10	Protected	III	813 (5/5)	17 (+7)
51200	0,5,10	Unprotected	III	545 (3/3)	18 (+8)

### Effect of azithromycin

We explored if a single dose of 2 g of extended-release azithromycin, given at the day of vaccination, can block development of (transient) asexual blood-stage parasitemia, as described above. Seven of the 14 volunteers (2 placebo and 5 vaccinees) of the 28-day regimen received azithromycin in addition to chloroquine prophylaxis at their first vaccination. All five who received PfSPZ developed transient asexual blood-stage parasitemia, although at a lower level, compared with chloroquine alone (*p* = 0.027, Kruskal–Wallis test on AUC) (Supplementary Fig. 2).

### Vaccination induces parasite-specific CD40L+CD4+CD45RO+T-helper cells with a pro-inflammatory signature

To profile T cell reactivity following vaccination with high sensitivity, antigen-reactive T cell enrichment^11^ (ARTE) was performed following in vitro iRBC or uRBC stimulation of PBMC. Thereby, the pro-inflammatory CD40L+TNF-α+IFN-γ+CD45RO+/CD4+T (Th1) memory helper-cell response and the stimulated naive T cell response (CD40L+TNF-α+IFN-γ+CD45RO–/CD4+) specific for Pf was monitored during the whole vaccination period. The frequencies of iRBC-reactive T cells were measured by flow cytometry. The gating strategy is summarized in Supplementary Figs. 3 and 4 and the calculation scheme to estimate the frequency of stimulated T cells is given in Supplementary Fig. 5.

**Fig. 2:**
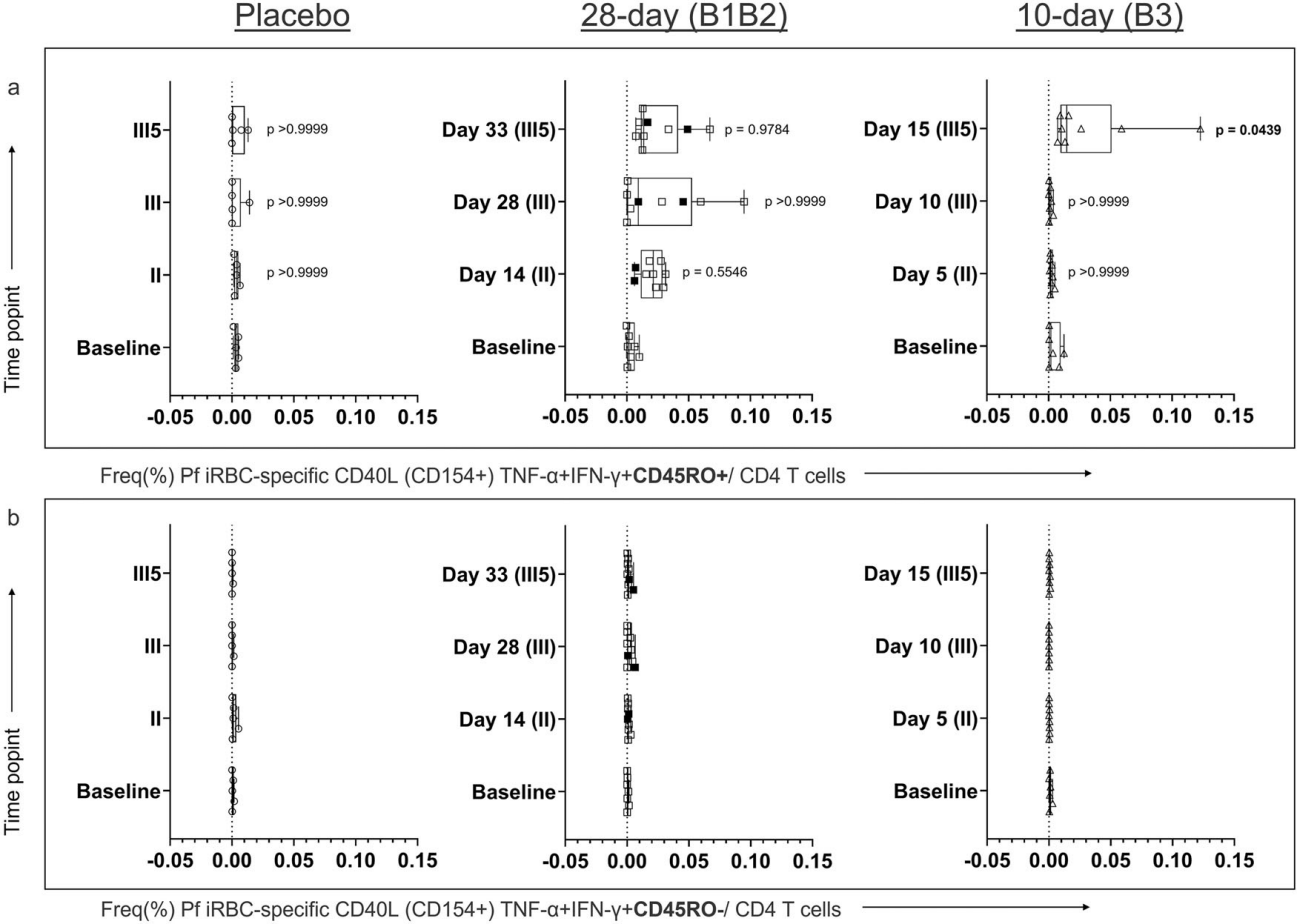
Ex vivo level of iRBC-specific polyfunctional memory and naive helper T cells (Th1). PBMCs were stimulated with iRBCs. Frequency of (**a**) memory Th1 cells (CD40L+CD4+TNF-α+IFN-γ+CD45RO+) and (**b**) naive Th1 cells (CD40L+CD4+TNF-α+IFN-γ+CD45RO–) were quantified by flow cytometry. Black filled squares represent the frequency of Th1 iRBC specific for two participants who did not develop detectable parasitemia after receiving the first immunization dose. Kruskal–Wallis test was used to assess the difference in the frequency of stimulated T cell population during the vaccination period. Statistical significance was considered when *p* < 0.05.

At baseline, iRBC treatment did not stimulate any memory Th1 or naive T-helper cells (Fig. 2). At day 14 (II, 28-day regimen) and day 15 (III5, 10-day regimen) respectively, the memory Th1 frequency increased only in vaccinated subjects, but not in the placebo group (Fig. 2a). The naive T-helper cells did not react to the iRBC stimulation after vaccination (Fig. 2b). Interestingly, two individuals of the 28-day regimen group who did not develop any parasitemia during the first immunization, also had the lowest frequency of iRBC-specific memory Th1 cells in that group (0.007 and 0.006%, median 0.021%), which is in the range of placebo (median 0.005%) and the 10-day vaccinee group at five days after the first immunization (median 0.003%) (Fig. 2, black dots). This observation indicates that in these individuals, iRBC-specific reactivity could not be induced by a single dose of PfSPZ alone and exposure to late liver stage/asexual blood stage eventually might be required. Despite this observation that these two individuals showed very low reactivity, the frequency of memory Th1 cells did not directly correlate with the overall exposure to parasites in the blood, represented by the calculated area under the curve of the parasitemia during the first immunization (Supplementary Fig. 6).

### PfSPZ–CVac induces iRBC-specific CD40L+CD4+pro-inflammatory Th1 cells one day before CHMI (C-1)

**Fig. 3:**
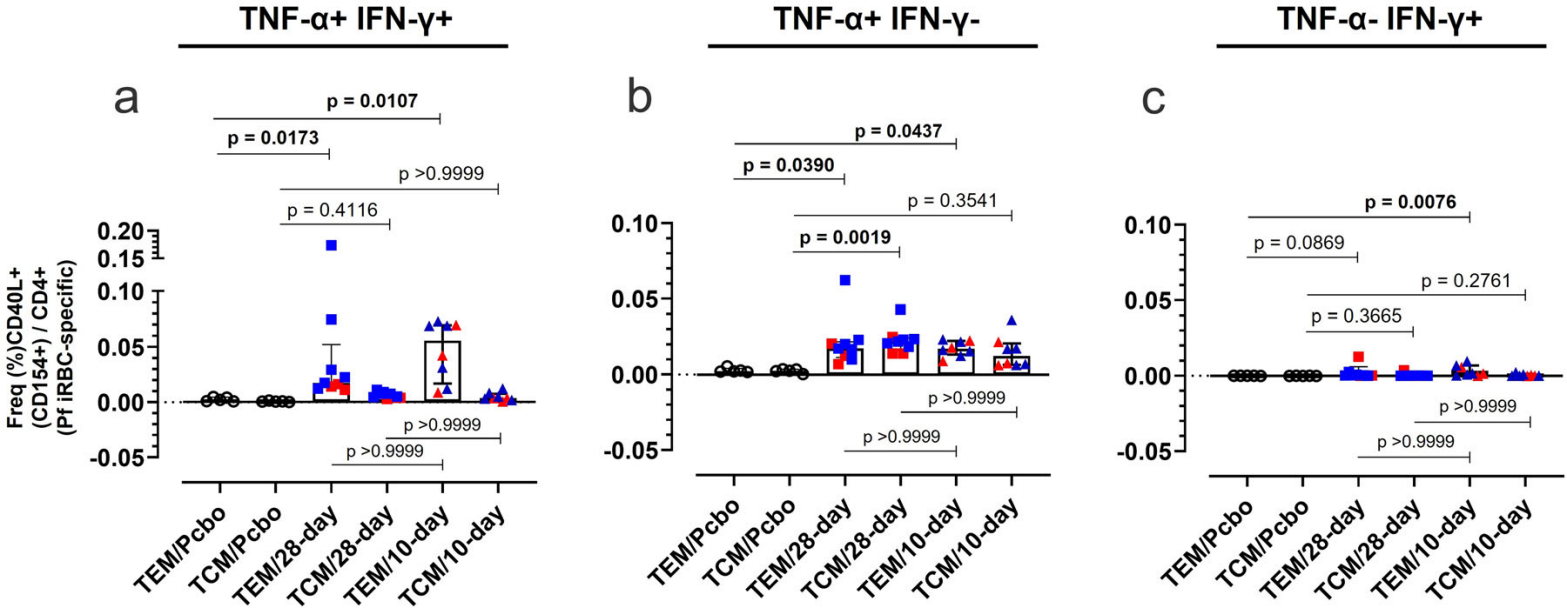
Pf-iRBC-specific activated mono- (TNF-α+ or IFN-γ+) and polyfunctional (TNF-α+ and IFN-γ+) effector memory and central memory CD4+T cells one day before CHMI. iRBC-specific cell frequencies obtained following ARTE are shown, comparing the influence of each regimen in the acquisition of circulating effector (TEM) or central (TCM) memory cells. (**a**) Polyfunctional T cells, (**b**) TNF-α+IFN-γ– monofunctional T cells, and (**c**) TNF-α– IFN-γ+ monofunctional T cells. Squares: 28-day regimen vaccinees; Triangles: 10-day regimen vaccinees; open circles: placebo. Red: no protection after CHMI; blue: protection after CHMI. Bar plots represent median and error bars show the interquartile range (IQR). The nonparametric Kruskal–Wallis with Dunn’s test is performed to analyze the differences between PfSPZ vaccinees (28-day, open squares and 10-day regimen, open triangles) and the nonimmunized volunteers (placebo, Pcbo). Differences between groups are considered statistically significant (bold) if *p* < 0.05.

Both immunization regimens (28-day and 10-day) significantly increased the frequency of polyfunctional effector memory (TEM, CD40L+TNF-α+IFN-γ+CD45RO+CD197–/CD4+) parasite antigen-specific T cells by day C-1 (Fig. 3). The 28-day vaccine regimen tended to generate about two times lower frequencies of polyfunctional TEM cells than the 10-day regimen, but the difference was not statistically significant. Also, TNF-α monofunctional TEM was significantly elevated in both regimens (Fig. 3b), and IFN-γ monofunctional TEM was only significantly elevated in the 10-day regimen (Fig. 3c).

Polyfunctional central memory cells (TCM, CD40L+TNF-α+IFN-γ+CD45RO+CD197+/CD4+) were similarly elevated in both the 10-day and the 28-day regimen after immunization as compared with the respective placebo controls (Fig. 3a). Only in the 28-day regimen, TNF-α monofunctional TCM was significantly elevated, all other estimated monofunctional TCM populations were elevated compared with the placebo control, although not statistically significant (Fig. 3b, c).

### ELISA shows similar IgG kinetics against the *P. falciparum* circumsporozoite protein (CSP)

**Fig. 4:**
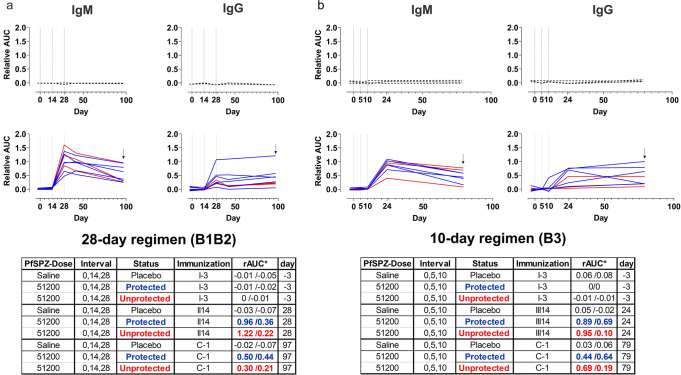
IgM and IgG ELISA titers against the malaria circumsporozoite protein (CSP). Analysis of IgM and IgG relative area under the curve (rAUC) against the CSP protein is represented for each vaccine regimen assessed. Sera were collected to perform ELISA (28-day = I-3, I14, II14, III14, and III69. 10-day = I-3, I5, II5, III14, and III69). B1B2 participants following 28-day regimen (**a**) and volunteers in B3 following the 10-day regimen (**b**). Blue lines describe IgM or IgG anti-CSP when vaccinees resulted protected after CHMI. Red lines by contrast, represent unprotected vaccinees and black dashed lines those who received placebo control (Pcbo). Vertical dotted lines indicate immunization days (0, 14, and 28 at 14-D interval and 0, 5, and 10 at 5-D interval). Arrows indicate the antibody levels against the CSP protein one day before challenge (C-1). Bottom tables summarize rAUC titers against CSP protein for each regimen, immunoglobulin isotype, status obtained after PfSPZ challenge (CHMI), and day of the study. (*) rAUC median per subgroup and immunoglobulin isotype backward slash separated (IgM (left)/IgG (right)).

IgG and IgM antibodies specific for the main surface protein of PfSPZ, CSP, were investigated in all volunteers by ELISA on each vaccination day, 14 days after the final boost and one day before CHMI. Both, CSP-specific IgG and IgM were negative at baseline in all participants. Participants following the 28-day regimen reached peak levels about 4 weeks after receiving the first vaccination (II14), and remained at similar levels until C-1 (Fig. 4a). This was unexpected since the third vaccination was expected to further increase the anti-CSP antibody levels. In the 10-day regimen group, the CSP-specific antibodies were not detectable before the last immunization on day 10, in 7 out of 8 individuals. In one individual, CSP-specific IgG could be detected just before the third immunization. In this group, antibody levels peaked by day 24 after the first vaccination (Fig. 4b). In parallel, the CSP-specific IgM response peaked after receiving the second or the third immunization dose following the 28- or 10-day regimen, respectively (Fig. 4). The IgM relative area under the curve was on average higher than the IgG response by II14 (28-day regimen, Fig. 4a) or III14 (10-day regimen, Fig. 4b), but declined between III14 and C-1. By contrast, the IgG levels remained at similar levels from III until C-1. Furthermore, differences between protected and unprotected vaccinees were not statistically significant at C-1 for any of the explored antibody isotypes. However, the anti-CSP IgG level was higher in protected (median relative area under the curve (rAUC) = 0.45) than in unprotected volunteers (median rAUC = 0.2) (Student’s *t*-test, two-sided, *p* = 0.073).

### Protein microarray

**Fig. 5:**
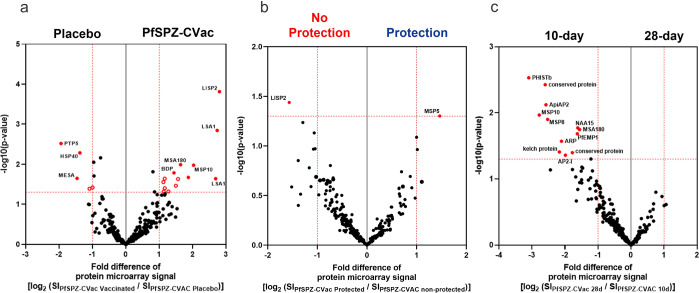
Screening of elicited humoral immune response using protein microarrays. Sera from all volunteers collected one day before CHMI (C-1) were assessed on protein microarrays containing 262 Pf proteins representing 228 unique antigens. Analysis was performed on C-1 data after subtraction of the individual background reactivity. Volcano plots represent the protein microarray data analyzed by the groups of the (**a**) antigen reactivity in vaccinated donors against placebo controls (immunogenicity), (**b**) reactivity of protected individuals against unprotected individuals, and (**c**) reactivity in the 28-day regimen vs. the 10-day regimen. Differentially recognized antigens (*p*-value <0.05 and fold change >2) are depicted in red. P- values are estimated using the two-sided Welch-corrected Student’s *t*-test.

The overall IgG antibody response against Pf was estimated by protein microarray in PfSPZ-exposed vaccinees (*n* = 9, 28-day regimen and *n* = 8, 10-day regimen), as well as in the placebo controls (*n* = 5) one day before CHMI (Fig. 5). The array comprises 262 Pf protein fragments representing 228 unique proteins as described before^18^.

IgG antibodies recognizing pre-erythrocytic malaria proteins such as the liver-stage antigen 1 (LSA1) and the liver-specific protein 2 (LISP2) were strongly induced in PfSPZ–CVac vaccinees. In addition, antigens associated with late liver stage and blood stage, like MSA180 and MSP10, were significantly elevated in the vaccinated group (Fig. 5a). Interestingly, the only antigen associated with protection in this study is MSP5, and antibodies specific for the liver-stage antigen LISP2 are even more elevated in the unprotected individuals (Fig. 5b).

Strikingly, when both vaccination regimens were compared, the parasite antigen-specific IgG antibody levels following the 10-day regimen were significantly higher than in the 28-day regimen (Fig. 5c). These antibodies were specific to several late liver-stage/early blood-stage antigens (PHISTb; Pf helical interspersed subtelomeric b, ApiAP2; Pf Apicomplexa plant-like transcription factor proteins, and MSP10; Pf merozoite surface protein 10).

## Discussion

Exposure to whole pre-erythrocytic-stage parasites has been one of the first successful vaccination approaches to malaria and at the moment seems still to be the most efficacious method to immunize against malaria in vertebrate hosts^19^. Seminal studies on human hosts receiving x-irradiated sporozoites by mosquito bite performed in the 1970s, showed that vaccination with attenuated sporozoites is safe and generates high antibody titers against the main surface protein of the sporozoite, the CSP protein^4^. Since then, the concept was reproduced with different modes of attenuation^20,21^ but it was only about twenty years ago that it was turned into an approach to develop a vaccine rather than being a proof-of-concept to develop other, mostly recombinant vaccines. The technical challenges of manufacturing and preserving billions of pharmaceutical-grade sporozoites for human use were overcome by the company Sanaria Inc.^22^ and clinical development was led by an international effort, including institutions from America, Europe, Asia, and Africa. In this framework, clinical trials have used whole sporozoites that were physically (PfSPZ Vaccine)^23–27^, chemically (PfSPZ–CVac)^10,18,28,29^ or genetically (PfSPZ–GAP1)^30^ attenuated in both malaria-naive and lifelong malaria-exposed human subjects.

The method and timepoint of attenuation of the parasites is important for achieving successful immunization, with killed sporozoites being not protective^31^ and strategies that allow full parasite development in the liver being the most effective^32^. One elegant way of achieving such late attenuation is the use of chemoprophylaxis based on a drug that kills asexual blood-stage but not liver-stage parasites. Proof-of-concept in mice^33^ and humans^34^ showed that this approach is highly effective.

Previously, we have translated this approach into a highly efficacious vaccine candidate using cryopreserved PfSPZ^10^. As the current immunization schedule is complex and therefore difficult to implement as a vaccine to be used in endemic regions and for travelers, we aimed at shortening and simplifying the regimen using CHMI as a measure of vaccine efficacy.

Healthy, malaria-naïve European young adults immunized with two regimen of three inocculations with fully infectious Pf sporozoites under chloroquine prophylaxis (PfSPZ–CVac) showing similar VE levels (28-day, 67% and 10-day regimen, 63%)^10^. Both regimens were safe and well tolerated in line with the previous 56-day regimen^10^ using the same PfSPZ dose. Recently, the 10-day regimen was assessed in US subjects and showed high VE (75%) using double number of PfSPZ per dose^35^. However, both 10-day regimens followed the same pattern regarding number and grade of AEs reporting more AEs after receiving the first vaccination shot at the time of peaking parasitemia. A subsequent trial conducted in Tübingen, allowed us to study a PfSPZ–CVac regimen partially inspired by the 10-day regimen with three immunizations of 1.1 × 10^5^ PfSPZ given at days 1, 6, and 29 under similar chloroquine dosage. Of interest, we achieved a high degree of heterologous protection (77% VE) challenging the vaccinees immunized using parasite strain PfSPZ NF54 of African origin with the genetically distant Pf 7G8 strain, which was isolated in South America^18^.

Volunteers receiving 5.12 × 10^4^ PfSPZ per dose developed adverse events related to the blood and lymphatic-system, including thrombocytopenia, lymphopenia, or leukopenia. General symptoms such as chills or fatigue were present in ~80% of all subjects. Mild nervous-system disorders such as dizziness or headache were similarly frequent. The use of twice the amount of PfSPZ per dose given at days 1, 6, and 29 further increased the likelihood of dizziness and headache^18^. In addition, the present study found an increase in both alanine and aspartate aminotransferase liver enzymes, which is consistent with the study published by Murphy et al.^35^ but not consistent with the study using the 56-day regimen^10^.

One participant allocated to chloroquine in the 28-day regimen became thick blood-smear positive following the first vaccination. At this time, he had no malaria symptoms but became symptomatic during treatment (Supplementary Fig. 2b). It is most likely that he did not swallow the chloroquine tablets as post hoc-measured plasma levels detected only traces of the drug (in agreement with a short exposure while keeping the tablets in the mouth). This shows that despite observed intake, parasite attenuation with an orally given drug has nonnegligible risks, which could lead to severe consequences when such an approach is used outside a tightly controlled clinical trial. Coformulation with PfSPZ or, at least, parenteral administration of the drug, would circumvent this problem. Chloroquine could have been given by intramuscular injection, but is not well tolerated and would require large volumes^36^. Intravenous injection is life-threatening, particularly when given as a bolus^37^.

The exploratory use of azithromycin in one of the two 28-day groups was done to assess if it can be used as an alternative to chloroquine but with a better adverse-event profile because the asexual blood stage could be omitted^16^ and its general good safety profile. Unfortunately, all participants developed asexual blood-stage parasitemia, albeit at lower levels (Supplementary Fig. 8). Thus, chemoprophylaxis in the two 28-day regimen groups (B1 and B2) was based on chloroquine alone, as per-protocol^10^. Nevertheless, the effect of azithromycin on parasitemia permits to hypothesize that a higher dose given either over a longer period or later during liver stage, could prevent blood-stage parasitemia. In a similar fashion, the use of pyrimethamine has recently shown that full protection is possible when the drug is given late during liver stage, preventing the asexual blood-stage development^38^.

In agreement with other PfSPZ–CVac studies involving malaria-naive PfSPZ vaccinees^10,18,34,35^, parasite-specific activated Th1 memory cells were equally expanded at C-1, irrespective of the regimen adopted, although the time between the third immunization and C-1 was key for the 10-day regimen vaccinees to catch up similar frequencies. Furthermore, we could associate the condensed regimen of 10 days with an elevated frequency of parasite-specific pro-inflammatory TEM cells, even though TEM cell number was not associated with protection against CHMI (Fig. 3).

Analysis of the IgM and IgG titers against the CSP protein, showed similar antibody anti-CSP profiles for both protected and unprotected participants, regardless of the vaccine regimen during the immunization phase (Fig. 4). Similarly, a higher number of PfSPZ per dose assessed in malaria-naive subjects following both the 56-day regimen^10,21^ and a shorter 10-day regimen^35^ did not change the pattern. Moreover, not only the dose, but also the use of pyrimethamine as PfSPZ–CVac partner drug instead of CQ has lately demonstrated a similar pattern^38^. The fact that in a 56-day regimen with lower PfSPZ number per dose (1.28 × 10^4^), there was no difference between protected (*n* = 6) and unprotected (*n* = 3)^10^ individuals, but in a study using higher dose of PfSPZ per dose (1.1 × 10^5^), a significant difference could be detected^18^, which may suggest a threshold for the anti-CSP IgG level that correlates with protection.

Furthermore, the antibody-response analysis performed after vaccination but before challenge (C-1) showed to be stronger following the regimen of 10 days compared with the 28 days (Fig. 5c). Consistent with the previous 56-day regimen performed in Tübingen using 5.12 × 10^4^ PfSPZ^10^, or the 28-day regimen (with immunizations at days 0, 5, and 28) using 1.1 × 10^5^ PfSPZ^18^ per dose, the antibody profile revealed an increased IgG reactivity against liver-stage markers such as LSA1 and LISP2 in all PfSPZ–CVac vaccinees (Fig. 5a). Moreover, vaccinees following the 10-day regimen showed 4–6 times higher IgG-reaction intensity towards Pf proteins associated with the liver-to-blood interface such as PHISTb, ApiAP2, or MSP10 than the 28-day regimen (Fig. 5c). This strong difference is surprising, but might be explained by the extended exposure of the immune system to liver-stage parasites of approximately 16 consecutive days, whereas the exposure to liver-stage parasites in the 28-day regimen is approximately 3 times 6.5 days with intermittent periods of blood-stage parasites, and a few days without any exposure to the parasite (Supplementary Fig. 9). Interestingly, the results presented here also show that MSP5 is strongly associated with protection after homologous PfSPZ Challenge. This protein is expressed in sporozoites, late liver stage, as well as the blood stage, and has been found to be immunogenic^10^. Likewise, it has been shown to be highly immunogenic in other vaccine trials using PfSPZ Vaccine before (Tumbo et al., in preparation). In agreement with it, the 28-day regimen (with vaccination at days 0, 5, and 28) using a higher number of PfSPZ per dose also revealed that protection against PfSPZ Challenge is strongly associated with another protein anchored in the merozoite surface protein 2 (MSP2). In the same way, MSP2 is associated with later stages of Pf infection in the liver^39^. Thereby, it is reasonable to believe that generating protective antibodies against two immunogenic antigens covering both initial and later phases of the pre-erythrocytic malaria such as CSP and MSP5, should offer a superior degree of protection against Pf.

Despite the interesting findings, the present work has some limitations affecting the extent of their conclusions. The sample size of the study is small. While this allows determination of clinically significant VE, it is difficult to determine immune parameters responsible for protection. Further studies should investigate, in addition to other parasite-specific CD4+ T cell subtypes, the contribution of the innate cell types (e.g., NK, NKT, and γ9δ2-T cells) to control parasite growth. In addition, immune response to whole sporozoites or to specific highly relevant antigens such as CSP might have revealed the role of single antigens in the overall immune response. Overlapping peptides might have been used to investigate specific CD4+ and CD8+ T cell response. For the future vaccine development, it also will be indispensable to extend the follow-up period to investigate the durability of protection.

In summary, study evidence shown here in addition to the findings by Murphy et al.^35^ highlights that it is safe to compress the PfSPZ–CVac regimen to ten days maintaining yet a high VE, which might be even further improved by increasing the number of PfSPZ number per dose.

## Methods

### TÜCHMI-002 Stage-B study design

This single-center, randomized, placebo-controlled, double-blinded phase-I/-II study was conducted from April 2015 to December 2015 at the Institute of Tropical Medicine in Tübingen, Germany. The PfSPZ formulation team was unblinded and thus was not involved in clinical or diagnostic activities.

Approval was obtained from the Ethics Committee of the Eberhard Karls University and University Clinics (EudraCT number 2013-003900-38, National Clinical Trial number (NCT): NCT02115516). The study was performed in accordance with Good Clinical Practice/International Conference on Harmonization guidelines. The sample size was calculated using the function nBinomial of the R package gsDesign^40^ which estimates the sample size required to detect a difference between two rates. According to study protocol, treatment allocation was random and blinded with a ratio of 2:1 (PfSPZ: placebo). A dedicated member of the formulation team, who was not involved in volunteer management or diagnostic activities, was responsible for keeping the randomization envelopes and dosing schedule, while a third party outside the study team and sponsor, generated and distributed the randomization list.

The primary efficacy endpoint was assigned to the proportion of volunteers parasitemic within 21 days after CHMI, while the secondary efficacy endpoint considered the time to detect parasitemia (prepatent period). The primary safety endpoint was set as the occurrence of related Grade-3 adverse events (AEs) from the first chemoprophylactic dose uptake (I-2) and PfSPZ challenge administration (I) until the end of the study. The secondary safety point focused on the appearance of any related AE from the time of the first administration of an immunizing regimen (PfSPZ–CVac) until the end of the study.

We recruited healthy, nonpregnant, malaria-naive volunteers aged between 18 and 45 years using the university email list.

Since the study was performed ambulatory, special safety precautions were applied to minimize risks of Pf malaria. To check the volunteers’ understanding of the trial, after providing informed consent, participants had to correctly fill out a questionnaire of ten questions about risks and obligations. If they failed their first attempt, volunteers had the opportunity to discuss the study and retry once. A second failed test led to study exclusion. Moreover, volunteers had to provide full contact details and name two contact persons, of which at least one had to live nearby and know about the participants’ whereabouts during the trial.

Out of the 46 volunteers screened, 21 were not eligible and 25 were enrolled and randomized to receive either 5.12 × 10^4^ PfSPZ Challenge of the NF54 strain or placebo (Supplementary Fig. 1).

Early conceptual design counted with three study groups, including two placebo controls each and randomization, was as follows: 5:2 participants for Group B1 (5.12 × 10^4^ PfSPZ: placebo), 5:2 participants for Group B2 (5.12 × 10^4^ PfSPZ NF54: placebo), and 9:2 participants for Group B3 (5.12 × 10^4^ PfSPZ NF54: placebo).

In Group B1, participants received 51,200 PfSPZ NF54 two weeks apart, using a chemoprophylactic regimen with CQ (10 mg/kg CQ base-loading dose, followed by weekly dosing with 5 mg/kg CQ base) for 6 doses.

Volunteers of Group B2 received the same PfSPZ dose two weeks apart and CQ chemoprophylaxis, but 2 g of extended–released azithromycin (ER-AZ) upon the first PfSPZ Challenge inoculation was also administered.

Volunteers of Group B3 received the most condensed immunization regimen with simultaneous administration of CQ and PfSPZ on days 0, 5, and 10 followed by an additional CQ dose on day 15.

Ten weeks after the last immunization, a challenge via CHMI was performed in all groups and individuals. CHMI was done by DVI of 3200 PfSPZ of the NF54 strain. Active follow-up of the participants was conducted 140 days after CHMI.

### TÜCHMI-002 Stage-B study-design modifications

Twenty-two healthy malaria-naive young adult volunteers out of 46 assessed for eligibility were chosen following a randomized double-blinded placebo-controlled immunization trial to explore and compare immunogenicity. In brief, volunteers were randomly distributed into two exploratory groups. The first one was the result of fusing previous B1 with B2 groups after azithromycin failure confirmed by qPCR data after the first PfSPZ DVI (I). Therefore, subjects receiving thereafter remaining two immunizations (II and III) fourteen days apart, remained as the 28-day regimen group (*n* = 14). There, 9/12 participants received PfSPZ Challenge under chloroquine (PfSPZ–CVac), while 3/12 were administered with placebo (NaCl 0.9%). The original B3 group remained as 10-day regimen group (*n* = 11, 9/11 receiving PfSPZ–CVac and 2/11 receiving placebo).

### Safety assessment

Safety was assessed through capture of adverse events (AE) (Supplementary Tables 2 and 3). The allocation ratio of 2:1 between immunized and placebo-treated volunteers was used to maximize the amount of safety/tolerability data related to PfSPZ Challenge with chemoprophylaxis. AE (and as part of these adverse reactions [AR]) was graded according to a predefined scale, whereas a study physician did the grading of unexpected AEs. Clinical AEs were elicited by spontaneous reporting and a defined set of open questions to improve discriminative capacity. Laboratory abnormalities were reported as AE when outside the reference range. The clinical team judged small deviations from the reference range as “non clinically significant”. Nevertheless, from day three after the “first immunization” (I-3) on, all laboratory abnormalities were regarded as AE. Laboratory-safety analyses were done before inoculation of the vaccine or administration of the loading dose (I-3, II-3, and III-3), one day before CHMI (C-1), on the day of the first parasitemia after CHMI for those who get positive, twenty-eight days after challenge (C28), and at any time the clinical team decided that a laboratory analysis was required. Due to the condensed immunization schedule of Group B3, the second and third laboratory-safety analyses were done on the day of PfSPZ Challenge inoculation.

### PfSPZ Challenge product

Cryopreserved NF54 PfSPZ was produced by Sanaria Inc. (Rockville, US). The parasites were isolated from infected mosquitoes bred under sterile conditions. Sporozoites were stored and transported in liquid nitrogen vapor phase at −140 to −196 °C. Formulation and reconstitution were made in Tübingen on the day of infection. Volunteers were inoculated within 20 min after thawing of PfSPZ.

### Parasitemia monitoring

qPCR and thick blood smears (TBS) were performed daily from 6 to 21 days after DVI according to the methods published elsewhere^10^. TBS were reviewed once daily by at least two delegated and trained members of the study team. Positivity was accepted in the case of concordant results. Antimalarial treatment (atovaquone/proguanil or artemether lumefantrin) was initiated in the case of TBS positivity.

### Sample collection

From all volunteers, heparinized whole blood was collected three days before the first immunization (I-3), before the second and the third immunizations (II and III), 5 days after the third immunization (III5), and one day before challenge (C-1). Sera were collected at days I-3, II, III, III14, and C-1 from all participants.

### Statistical analysis

Efficacy analyses were performed on the per-protocol (PP) population. Primary efficacy was calculated using the unconditional exact Boschloo test, vaccine efficacy was expressed as (1 minus relative risk) x100%. Survival analysis was performed by using the log-rank test and plotted with inverse Kaplan–Meier curves. Malaria positivity was accepted when qPCR detected ≥100 parasites per milliliter of blood. A *p*-value < 0.05 deemed to indicate statistical significance. Statistical analyses were performed under R version 3.1.2.1 with exact2x2 and rms packages. All statistical tests were performed two-sided.

### Antigen-reactive T cell enrichment (ARTE)

Peripheral blood mononuclear cells (PBMCs) were obtained by ficoll-paque plus density gradient centrifugation (GE Healthcare Life Science, ref# 17-1440) from the heparinized whole blood. All assays were performed using fresh PBMC samples.

In brief, PBMCs from each participant were placed in RPMI 1640 medium (Sigma-Aldrich), supplemented with 5% (v/v) AB serum (Lonza) and 2 mM L-glutamine (PAA Laboratories) for 18 h at 37 °C at 5% CO2. Hence, in order to enrich the Ag-specific T cells, plate wells containing 1 × 10^7^ PBMCs were stimulated for 5 h with the following stimulants: (a) negative control; 500 μl of thawed uninfected red blood cells (RBC) from a culture collection aliquoted and stored at −80 °C, (b) 500 μl of a thawed aliquot containing infected red blood cells (iRBCs, Pf3D7 laboratory strain; 90% schizonts by microscopy), and (c) 5 µg/ml of Staphylococcus endotoxin B (SEB, Sigma-Aldrich; ref# S4881) as a positive control. All samples reached a final volume of 1500 μl/well.

In addition to the stimulants cited above, all samples were incubated in the presence of 1 µg/ml anti-CD40 (#Cat 130-094-133) and 1 µg/ml anti-CD28 (#Cat 130-093-375) functional-grade pure antibody (Ab) (both Miltenyi Biotec).

Brefeldin A (Sigma, #Cat B7151) was employed at 1 µg/ml for 2 h to stop cytokine release. As a negative control, nonautologous uninfected RBCs (uRBCs, type O+) obtained from the blood- donation center (Zentrum für Klinische Transfusionmedizin Tübingen gemeinnützige GmbH) were used for each participant.

After incubation, a small fraction of 30 µl from the original sample (ORI) was taken to assess the original phenotyping before magnetic separation by direct staining with 65 μl of the original fraction staining mix. The leftover sample was centrifuged at 300 × *g* for 5 min. The pelleted cells were labeled with 10 μl of anti-CD40L-Biotin (Miltenyi Biotec, #Cat 130-092-658) for 10 min at 4 °C. Samples were subsequently labeled using the Anti-Biotin MicroBeads (#Cat 130-090-485) and washed two times with PEB buffer (1xPBS, 2 mM EDTA, and 0.5% BSA) before being loaded into the magnetic columns to perform the anti-biotin magnetic sorting separation (MACS) (Miltenyi Biotec, #Cat 130-042-201).

Once the columns had retained the coated cells, 60 μl of the staining mix for the surface staining was added to each column. Following, 1xPBS (Gibco, Life Technologies) was used two times before eluting the sample with 500 μl of PEB. The eluted cells were collected in 1.5-ml tubes (Eppendorf) to be fixed with 200 μl of Fixable Live/Dead (Inside stain kit, Miltenyi Biotec, 1:100).

A second round of magnetic separation was performed over the fixed suspension using a new set of columns. Previously, a rising step with 500 μl of PEB was done to prepare the new columns.

Once the fixed positive fraction was retained again, the columns were washed out with 500 μl of PEB before adding 200 μl of Inside Perm buffer (Inside stain kit, Miltenyi Biotec, #Cat 130-090-477) into the column. Then, 60 μl of the intracellular staining cocktail was added to every column. After 15 min of incubation at room temperature, the columns were washed out with 200 μl of Inside Perm buffer. Eluted cells with 1000 μl of PEB buffer were collected into 1.5 ml of Eppendorf tubes to be centrifuged at 300 g for 5 min. The pellet was resuspended in 200 μl of PEB buffer to flow cytometry analysis. If not mentioned otherwise, the monoclonal antibodies were purchased from Miltenyi Biotech.

#### Original (ORI) staining

About 5 μl of the following monoclonal antibodies plus 35 μl of PEB were used per sample. Monoclonal antibodies: anti-CD8-PerCP (#Cat 130-008-057, 1:13), anti-CD14-PerCP (#Cat 130-098-072, 1:13), anti-CD20-PerCP (#Cat 130-098-097, 1:13), anti-CD4-VioBlue (#Cat 130-099-683, 1:13), anti-CD45RO-PE-Vio 770 (#Cat 130-099-692, 1:13), and anti-CCR7-FITC (UCHL-1, Becton Dickinson Biosciences, 1:13).

#### Positive-selection (PS) staining

About 6 μl of the following monoclonal antibodies plus 20 μl of 1xPBS were used per sample to stain the surface of the CD40L+−T cells: anti-CD8-VioGreen (#Cat 130-096-902, 1:10), anti-CD14-VioGreen (#Cat 130-096-875, 1:10), anti-CD20-VioGreen (#Cat 130-096-094, 1:10), anti-CD4-APC-Vio770 (#Cat 130-100-457, 1:10), anti-CD45RO-FITC (#Cat 130-095-462, 1:10), and anti-CCR7-PE (#Cat 130-099-361, 1:10). Permeabilized samples were stained with 6 μl of anti-CD40L-VioBlue (#Cat 130-096-217, 1:10), in combination with markers for the following cytokines: anti-TNF-α-PE-Vio 770 (6 μl) (#Cat 130-096-755, 1:10), and anti-IFN-γ-PerCP 5.5 (0.6 μl) (Biolegend, #Cat 502526, 1:100)

### T cell monitoring data analysis

After PBMC isolation (step 0), 10^7^ cells were placed in three separate wells. Each well was stimulated with the uninfected red blood cells (uRBC), the Pf*-*infected red blood cells (iRBCs, 90% Schizont), or the Staphylococcal enterotoxin B (SEB) at each timepoint. In step 1, 30 μl of each stimulated sample (original sample, ORI) is used to calculate the number of CD4+ T cells stimulated with uRBCs, iRBCs, or SEB (Supplementary Fig. 5). The remaining sample of each well is magnetically isolated to enrich the CD40L+ population stimulated with uRBC, iRBC, or SEB (step 2). The resulting positive samples (PS) are analyzed by flow cytometry for the intracellular staining of TNF-α and IFN-γ. Moreover, each subpopulation of mono- or polyfunctional T cells was classified into effector memory T cells (TEM), central memory T cells (TCM), and naive T cells (TN) using the antibodies anti-CD45RO and anti-CCR7 (Supplementary Figs. 3 and 4).

In step 3, the net percentage of iRBC-specific CD40L+CD4+ T cells is calculated according to the formula (Equation 1, Supplementary Fig. 6).

*Equation 1: Calculation of net percentage of iRBC-specific CD40L*+*CD4*+ *T cells using the ARTE*

$${{{\mathrm{net}}}}\,{{{\mathrm{fraction}}}}\,{{{\mathrm{iRBC}}}}\,{{{\mathrm{specific}}}}\,{{{\mathrm{TEM}}}} = \frac{{{{{\mathrm{iRBC}}}}\,{{{\mathrm{stimulated}}}}\,{{{\mathrm{TEM}}}}\,{{{\mathrm{events}}}} - {{{\mathrm{uRBC}}}}\,{{{\mathrm{stimulated}}}}\,{{{\mathrm{TEM}}}}\,{{{\mathrm{events}}}}}}{{{{{\mathrm{fraction}}}}\,{{{\mathrm{of}}}}\,{{{\mathrm{iRBC}}}}\,{{{\mathrm{stimulated}}}}\,{{{\mathrm{Th}}}}\,{{{\mathrm{cells}}}} \times 10^7\,{{{\mathrm{Total}}}}\,{{{\mathrm{stimulated}}}}\,{{{\mathrm{PBMCs}}}}}} \times 100\%$$

Cells were measured using the BD FACS Canto II. FlowJo V10 was used to analyze flow cytometry data. Both statistical analysis and figures were generated using GraphPad Prism 8.1.2 (GraphPad Software, San Diego, CA) and FACSDiva software (BD Biosciences).

### ELISA

IgM and IgG antibodies directed against recombinant PfCSP were measured via ELISA, as described previously^41^ although including some modifications.

High-binding 96-well plates (Corning Costar 96-Well EIA/RIA Plates) were coated overnight at 4 °C with 20 ng/well of PfCSP diluted in 100 µl/well of 0.1 M sodium bicarbonate buffer (pH 9.6). Plates were washed three times using 0.1% Tween 20 in 1 X PBS buffer and blocked with 200 µl/well of blocking buffer containing 5% bovine serum albumin, 0.1% Tween 20, and 0.5 mM EDTA in 1 X PBS buffer. Following 3 h of incubation at room temperature on an orbital shaker, the plates were washed three times. Samples were added as unique in twofold serial dilutions of plasma in 100 µl/well of blocking buffer covering a dilution range from 1:400 down to 1:51,200.

On every plate, positive- and negative-control sera were included and equally diluted. Plasma of a naive European donor served as negative control. Pooled plasma derived from vaccinees of a different PfSPZ immunization study (MAVACHE day III14, verification phase, NCT02704533) was used as positive control.

Plates were incubated for 1 h at room temperature on the shaker before being washed three times. Secondary antibodies (Peroxidase-conjugated AffiniPure Goat anti-human IgG Fc fragment, Jackson ImmunoResearch) were added in a concentration of 0.088 µg/ml in 100 µl/well of blocking buffer and plates were incubated for 1 h at room temperature on the shaker. Following three washes, 100 µl/well of TMB peroxidase substrate was added and plates were incubated for approximately 10 min in the dark. The reaction was stopped by adding 50 µl/well of 1 M hydrochloric acid. Optical densities (OD) were determined in the CLARIOstar microplate reader (BMG LABTECH) at 450 nm and 620 nm.

### ELISA data analysis

Data analysis was done in R version 3.6.2 using the packages “drc” (Ritz, Baty et al., 2015), “DescTools” (Signorell et al., 2018), “tidyr” (Wickham, Lionel, 2018), and “ggplot2” (Wickham, 2016). Signals were corrected for their backgrounds by subtracting the ODs measured at 620 nm from the ODs measured at 450 nm. For every control and sample, a four-parameter logistic curve was fitted using the common logarithm of the dilution factor (400–5.12 × 10^4^) as x- and the measured ODs as y-values. The area under the curve (AUC) was calculated and related to the AUC of positive and negative control on the corresponding plate that were set to 1.0 or 0.0, respectively (Equation 2).

*Equation 2: Calculation of relative relative area under the curve (AUC) for measurement of ELISA*

$${{{\mathrm{AUC}}}}_{{{{\mathrm{relative}}}}\,({{{\mathrm{rAUC}}}})} = \frac{{{{{\mathrm{AUC}}}}_{{{{\mathrm{test}}}}\,{{{\mathrm{sample}}}}}-{{{\mathrm{AUC}}}}_{{{{\mathrm{negative}}}}\,{{{\mathrm{control}}}}}}}{{{{{\mathrm{AUC}}}}_{{{{\mathrm{positive}}}}\,{{{\mathrm{control}}}}}-{{{\mathrm{AUC}}}}_{{{{\mathrm{negative}}}}\,{{{\mathrm{control}}}}}}}$$

### Microarray assay

Protein microarrays to assess antibody reactivity against Pf antigens have been performed according to optimized procedures^42–44^. Microarrays were produced as described previously at the University of California Irvine, Irvine, California, USA^45^. In total, 251 Pf proteins were expressed using an *Escherichia coli* lysate in vitro expression system and spotted on a 16-pad ONCYTE AVID slide, representing 212 important Pf antigens. All antigens spotted on the array have been published before^18^. Secondary antibodies (goat anti-human IgG Qdot®800) were obtained from Grace Bio-Labs Inc. (Bend, OR).

Plasma was withdrawn from all study participants one day before challenge (C-1) by phlebotomy and stored at −80 °C. Plasma samples were diluted 1:100 in 0.05X Super G Blocking Buffer (Grace Bio-Labs Inc.) containing 10% *E. coli* lysate (GenScript, Piscataway, NJ) and incubated for 30 min on a shaker at room temperature (RT). Meanwhile, microarray slides were rehydrated using 0.05X Super G Blocking buffer at RT. Subsequently, rehydration buffer was removed, and samples added onto the slides. Samples were incubated for 2 h at RT on a shaker (180 rpm). Afterward, diluted plasma samples were removed, and microarrays washed using 1X TBST buffer (Grace Bio-Labs, Inc.). Secondary antibodies were applied at a dilution of 1:250 and incubated for 2 h. After a final washing step, slides were dried by centrifugation at 500 g for 10 min. Slide images were taken in a ArrayCAM® Imaging System using the ArrayCAM 400-S Microarray Imager Software (Grace Bio-Labs Inc.).

### Microarray data analysis

Microarray data were analyzed using the R statistical software package version 3.6.2. All images were manually checked for any noise signal. Image quality was very high, but rare blurry spots were removed from further analysis. Spot signals were corrected for local background reactivity by applying a normal–exponential convolution model^46^ using a saddle-point approximation for initial parameter estimation^47^ (available in the limma package^48^ v3.28.14). Data were then log2-transformed to approach a normal distribution of spot signals. Interarray normalization reducing the effect of sample-specific background reactivity to *E. coli* antigens contained in the spots' matrix was performed by subtracting the median signal intensity of mock expression spots on a particular array from the actual spot signals measured on the same array. Differential recognition of antibodies in the different study outcome-groups was analyzed by Student’s t-test, and the respective *p*-value and fold-change differences of antibody-level means were given. Heatmaps, boxplots, and volcano plots were generated using the gplots, ggplot, and PAA packages, respectively.

### Reporting summary

Further information on research design is available in the Nature Research Reporting Summary linked to this article.

## Supplementary information

SUPPLEMENTAL MATERIAL

REPORTING SUMMARY

## Data Availability

All data supporting the findings of this study are available upon reasonable request from the corresponding author.

## Code availability

The code used for data analysis is available upon reasonable request from the corresponding author and Rolf Fendel (rolf.fendel@uni-tuebingen.de). For statistical analyses, R statistical software package version 3.6.2 and GraphPad Prism 8.1.2 (GraphPad Software, San Diego, CA) was used.
