# Supplementary material for: Efficacy, T cell activation and antibody responses in accelerated Plasmodium falciparum sporozoite chemoprophylaxis vaccine regimens
Source: NPJ Vaccines. 2022 May 31;7:59. doi: 10.1038/s41541-022-00473-1 (PMC9156686; doi:10.1038/s41541-022-00473-1)
Supplement: Supplementary file 1 — SUPPLEMENTAL MATERIAL [file 41541_2022_473_MOESM1_ESM.pdf]

Supplementary information

Supplementary Figure 1

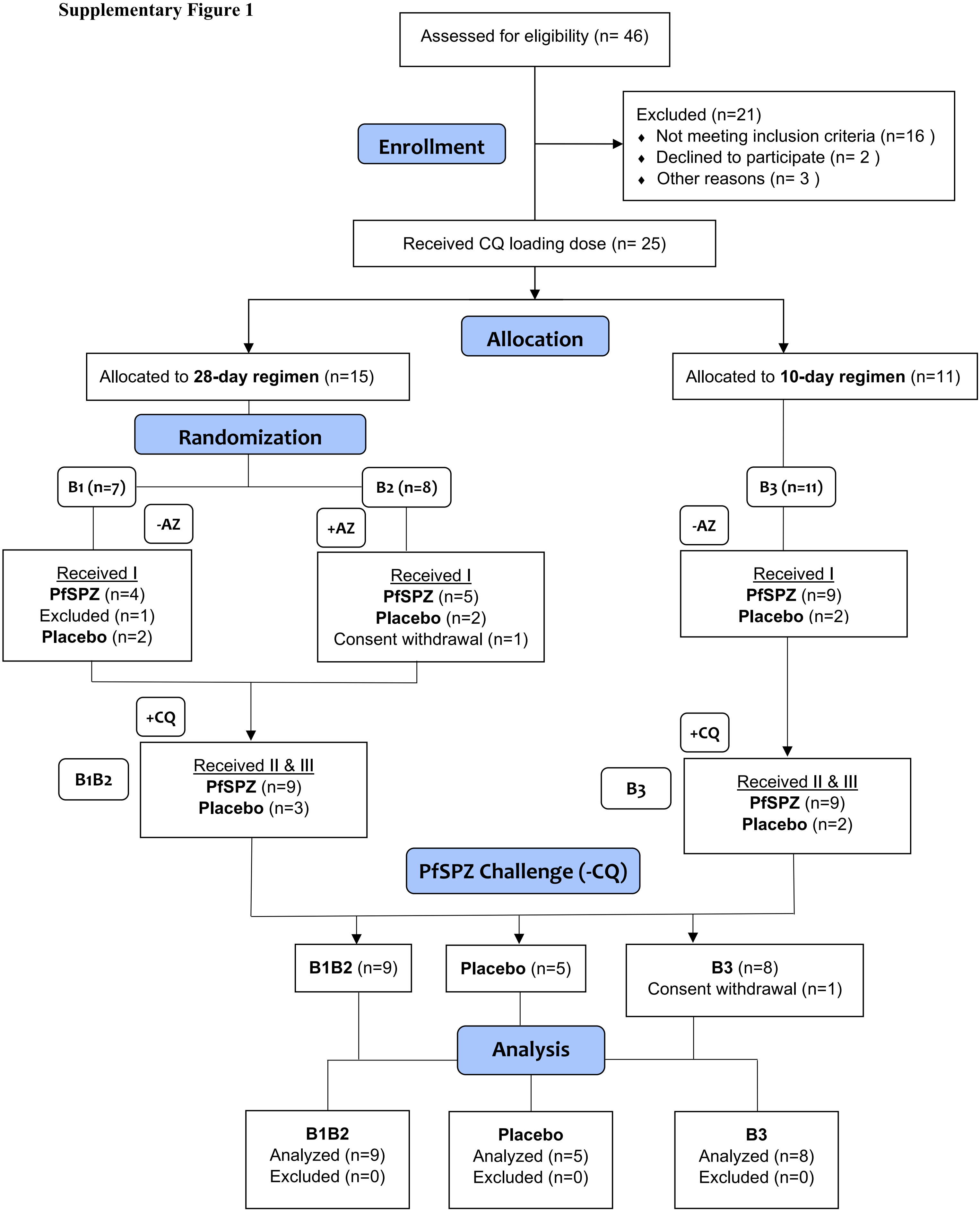

Supplementary Fig.CONSORT diagram. Flow chart shows every individual step of the randomized controlled vaccination trial (enrollment, allocation, follow-up, and analysis).

Supplementary Figure 2

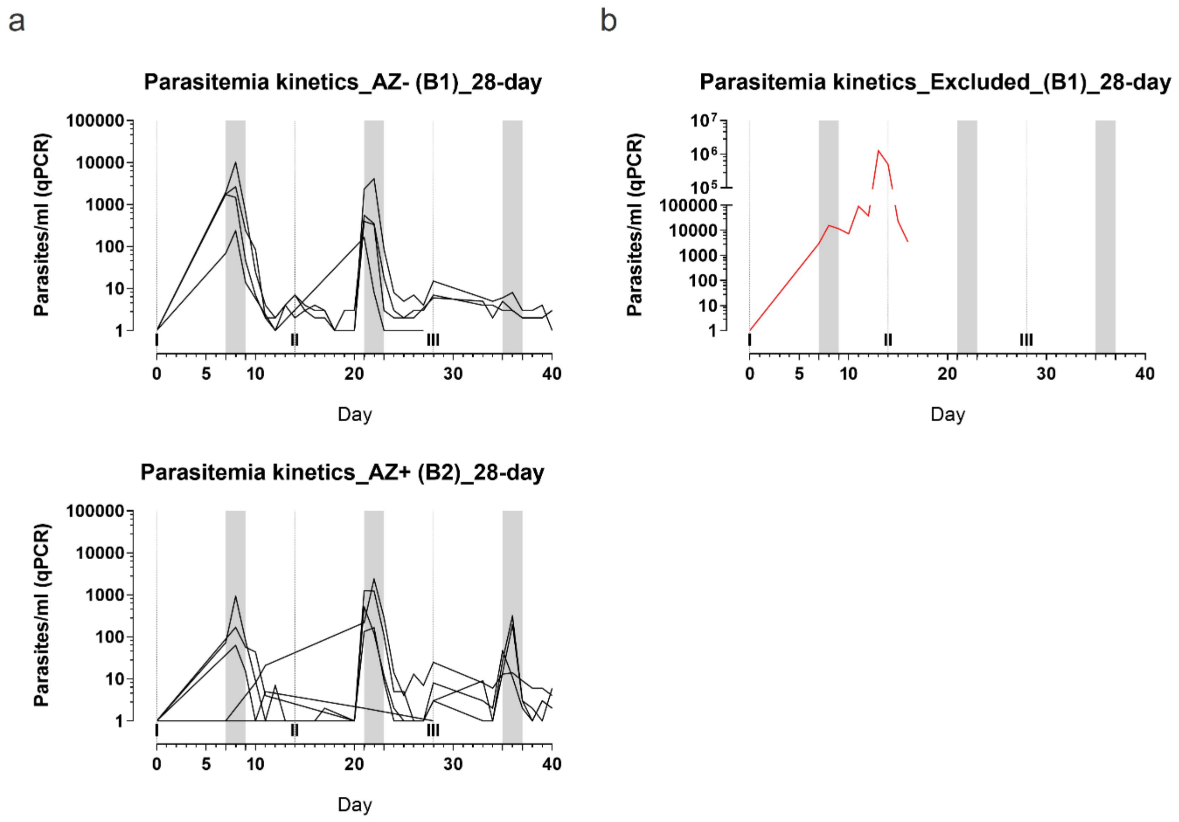

Supplementary Fig. 2. **Parasitemia kinetics for early 28-day regimen subgroups B1 (only treated with chloroquine, CQ) and B2 (CQ + Zithromax Uno (AZ))**.

a. Parasitemia detected and quantified by quantitative PCR in B1 participants (median: 2066, n=5) receiving 10 mg/Kg of CQ as loading dose two days before first PfSPZ-CVac immunization plus an extra dose of 5 mg/Kg of CQ seven days after it (day 5 of the study), are graphically compared at peaked times (day 8) with B2 participants (median: 63, n=5) receiving 10 mg/Kg CQ loading dose (I-2) plus 2 g azithromycin the same day the second immunization dose is administered (I14). Parasitemia levels from day 7 until receiving second immunization (I14=II) are plotted in b. Red line highlights parasite kinetics in the blood stream of the participant excluded from the study **(B1 subgroup. Supplementary Fig.1)**.

Supplementary Figure 3

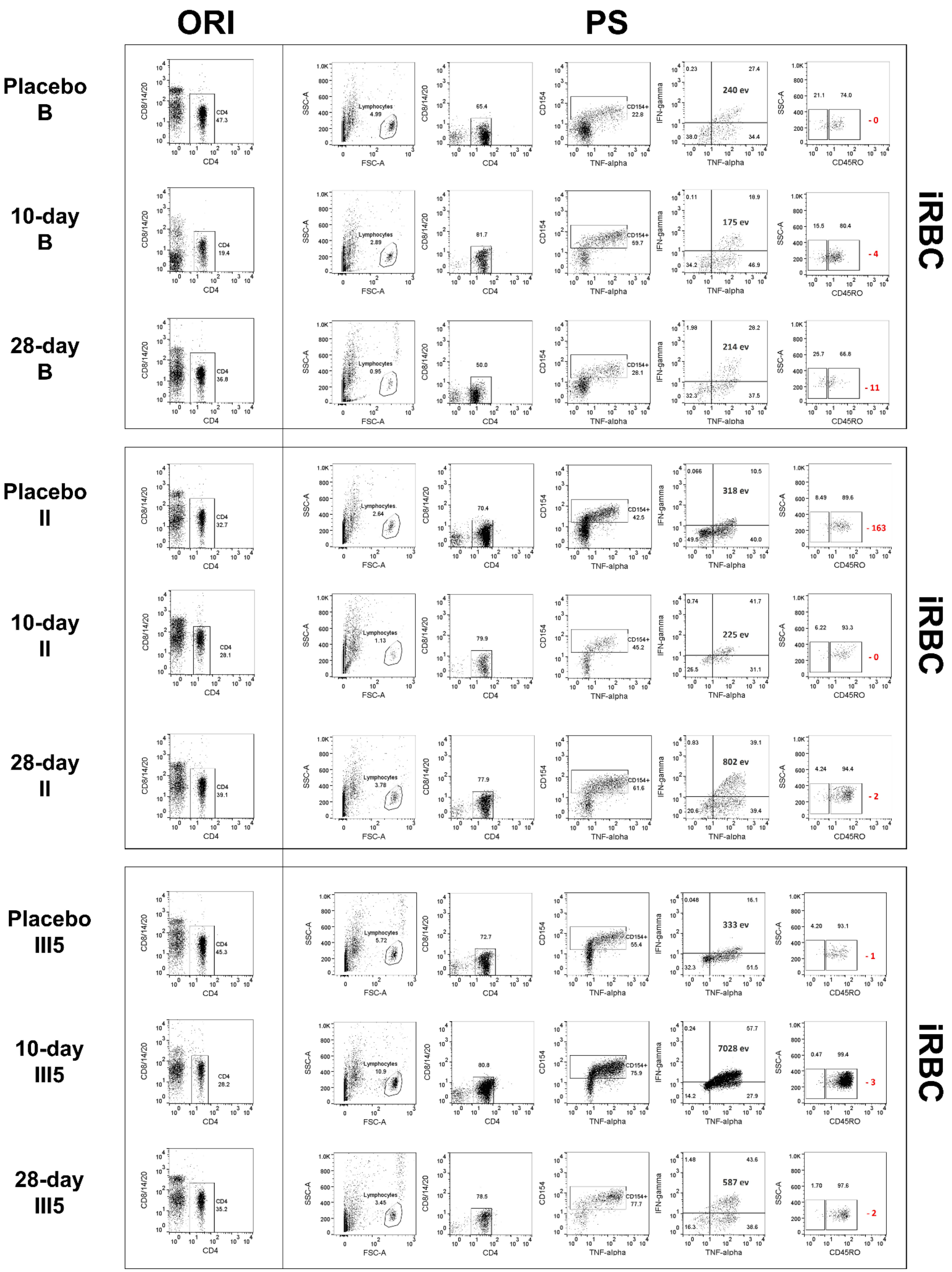

Supplementary Fig. 3. Pf iRBC-specific CD40L (CD154+) TNF-α+IFN-γ+ CD45RO+/- /CD4+ T cell layout

The layout offers a synthetical overview about the flow cytometry analysis involved in the ARTE calculations following the monitoring of iRBC-specific CD40L (CD154+) TNF-α+IFN-γ+ CD45RO+/- /CD4+ T cell along the immunization phase seen in Fig. 2. ORI plots stand for the CD4+ T cell stimulated and PS for the events CD40L (CD154+) TNF-α+IFN-γ+ iRBC-specific. Numbers in PS iRBC-specific CD40L (CD154+) TNF-α+IFN-γ+ permit to interpret the changes depicted in Fig.2.Red numbers in the CD45RO+/- plots indicate the uRBC event count subtraction for the CD45RO+ population required to calculate the Pf iRBC-specific CD40L (CD154+) TNF-α+IFN-γ+ CD45RO+/CD4+ T cell net frequency according to ARTE principle shown in Supplementary Fig.6.

Supplementary Figure 4

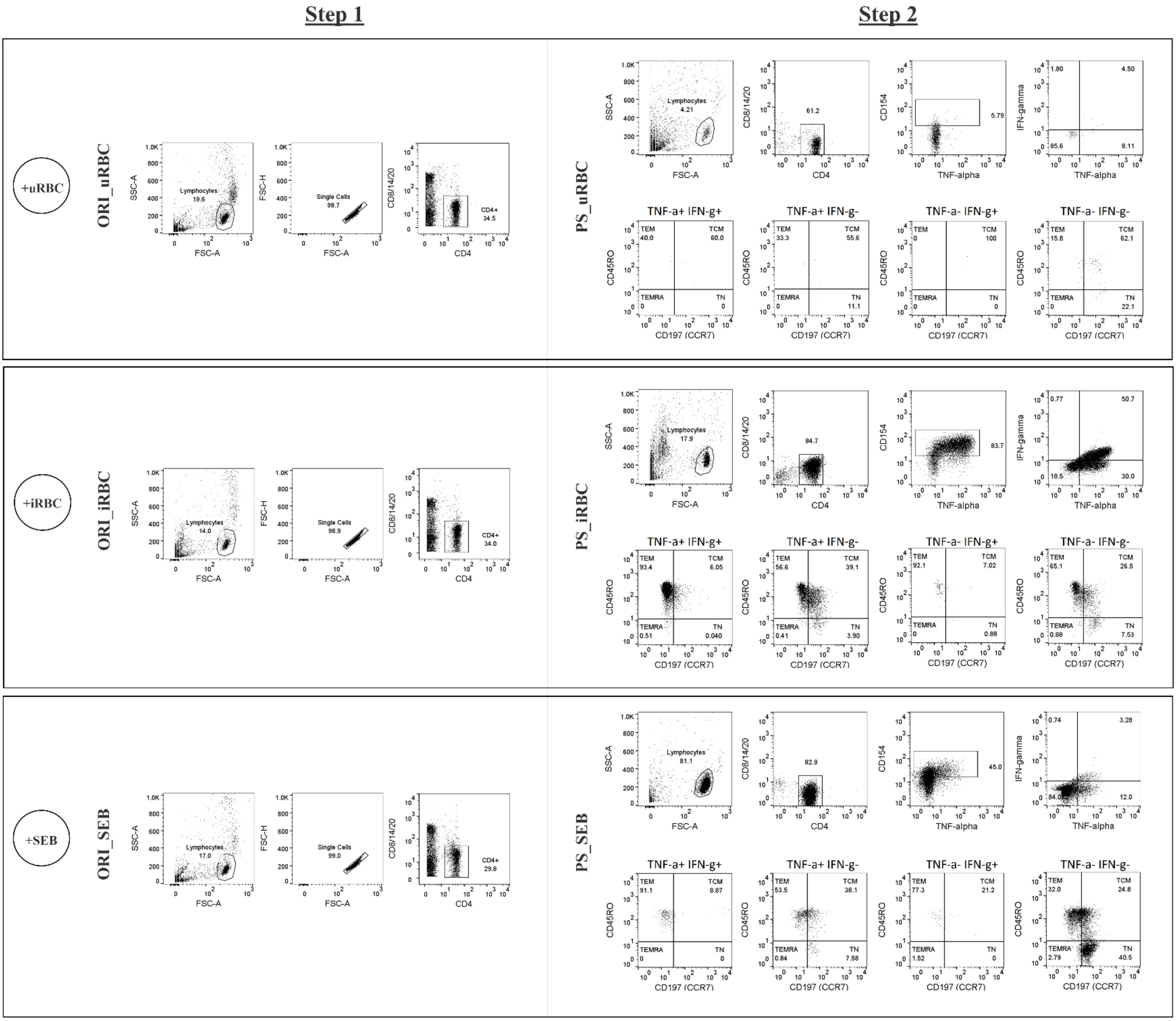

Supplementary Fig. 4. **Antigen Reactive T-cell Enrichment (ARTE) overview**.

A schematic view about the ARTE methodology showing the flow cytometry dot plots involved in the evaluation of the reactivity and specificity against the antigen of interest (iRBC) in a random participant at any time point. uRBC stands for uninfected red blood cells (negative control), iRBCs for *Plasmodium falciparum* (Nf54 strain) infected red blood cells, and SEB for Staphylococcal enterotoxin B (positive control). In Step 1 the original sample (ORI) establish the total CD4+ T cells stimulated for each stimulant. After two series of magnetic isolation in step 2, the positive selected (PS) enriched CD40L+CD4+ cluster is deeply analyzed regarding their intracellular cytokines TNF-α and IFN-γ and the extracellular CD45RO and CC197 (CCR7) memory markers.

Supplementary Figure 5

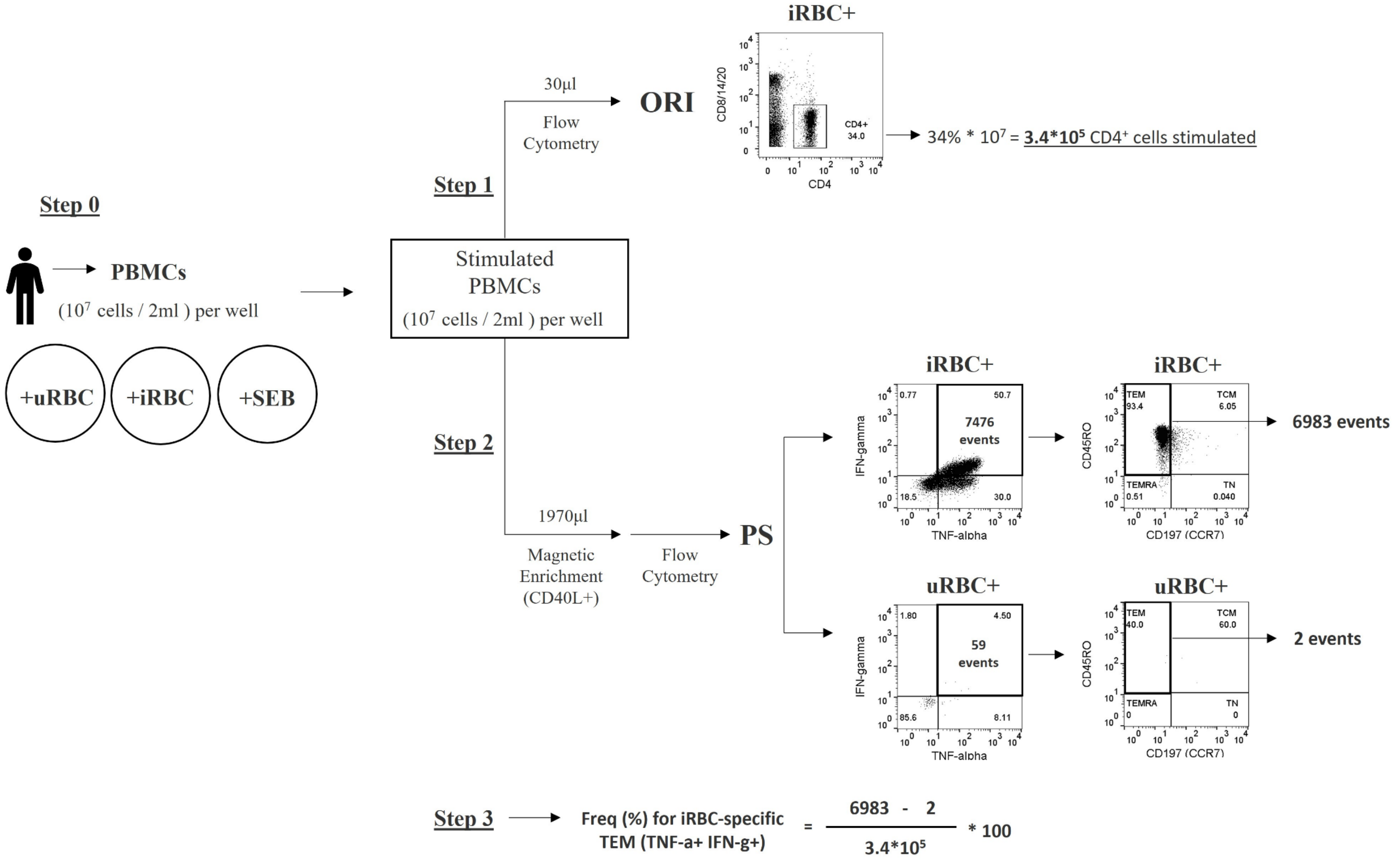

Supplementary Fig. 5. . ARTE frequency calculation.

Heparinized blood from study participants were collected to isolate the peripheral mononuclear cells (step 0). Every three independent wells received 10^7 cells in 2ml of culture medium each. Samples were stimulated separately with three different stimulants, the negative control; uninfected red blood cells (uRBCs), the *Plasmodium falciparum* infected red blood cells (iRBCs; 90% Schizonts) and the positive control; the Staphylococcal enterotoxin B (SEB). In addition, all samples were incubated in the presence of CD40 and CD28 grade pure antibody (Step 1). After stimulation, 2 ml sample/well was split in two. 30µl of the original sample (ORI) were used to estimate the number of CD4+ T cells stimulated with uRBC, iRBC or SEB (Step 2). The remaining 1970µl were used to enrich the CD40L+ population by magnetic separation. The resultant positive sample (PS) was analyzed by flow cytometry (Step 2). Down, a representative calculation for the frequency of the T effector memory (TEM) CD40LCD4 (TNF-α+ IFN-γ+) cell frequency is shown (Step 3).

Supplementary Figure 6

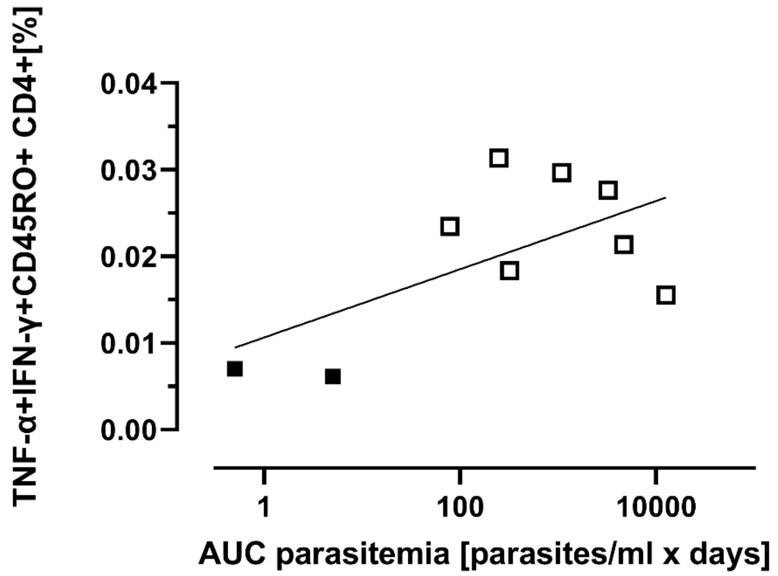

Supplementary Fig. 6. Dependance of Th1 memory response on the previous parasite exposure. Parasitemia was estimated by PCR and the overall area under the curve (AUC) parasitemia over the first peak of parasitemia between day 6 and 10 after immunization was estimated. In addition, memory Th1 cells (TNF-α+IFN-γ+CD45RO+ CD4+) were estimated using the ARTE method (Pearson correlation coefficient r = 0.6162, p = 0.077)

Supplementary Figure 7

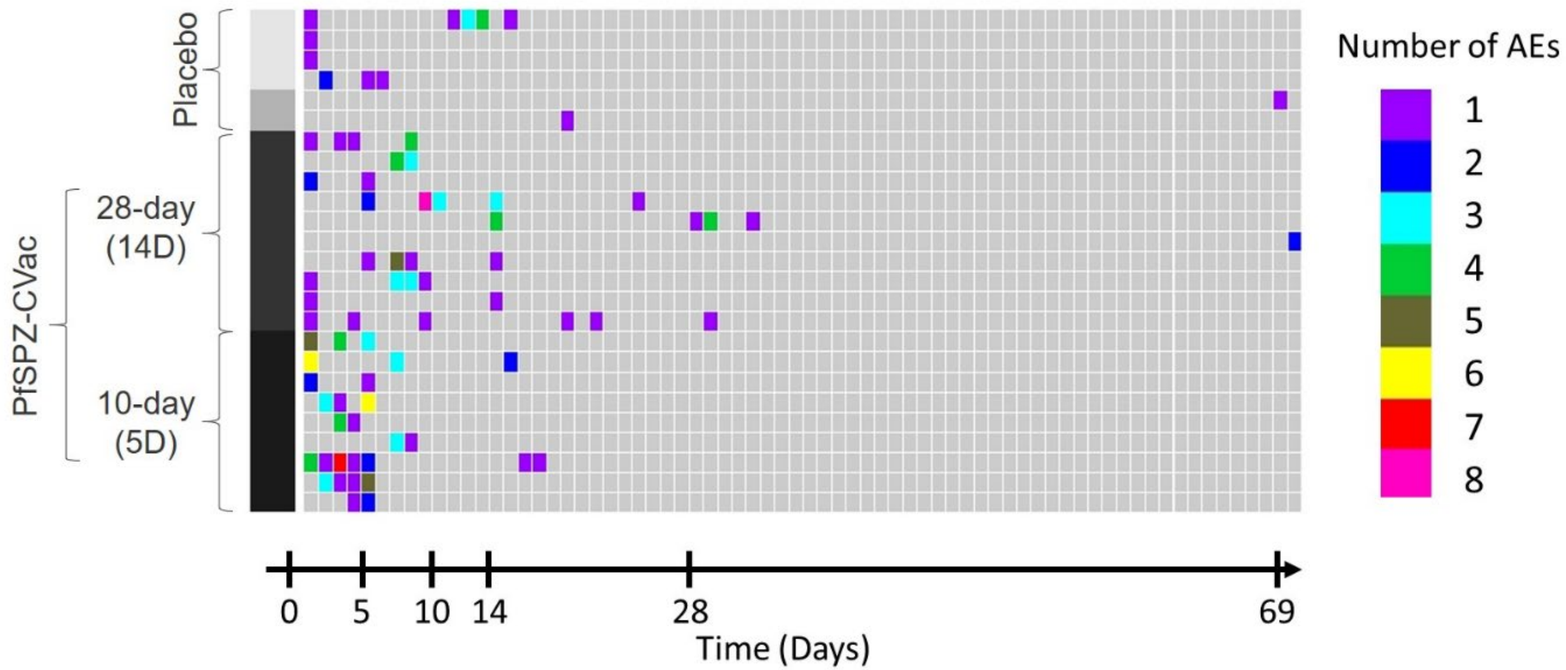

Supplementary Fig. 7. **AEs during the immunization period are recorded for every participant**. The total number of mild and moderate AEs per individual (rows) and day (columns) are plotted as a heatmap. The study population is divided at PfSPZ-CVac treated and not treated (Placebo) level and PfSPZ-CVac sublevel, discriminating between the 28-day regimen (14D interval) and 10-day regimen (5D interval). Numbers of AEs are represented as given by the color scheme in the legend. AEs on Day 1 occurred after administration of fully infectious Pf sporozoites (Nf54 strain) under chloroquine loading dose treatment reached two days before receiving first immunization.

Supplementary Figure 8

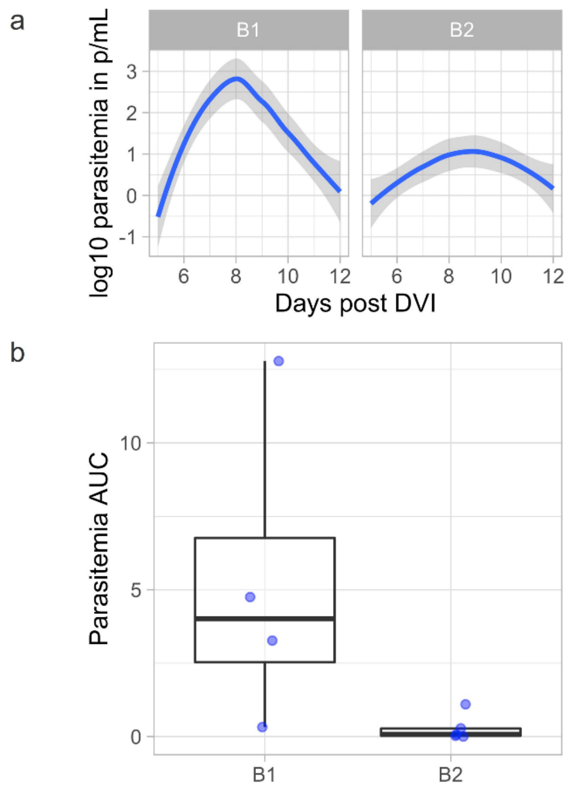

Supplementary Fig. 8. **Azithromycin effect on liver to blood inoculum**.

Parasitemia peaks 7-9 days after first intravenous immunization with $5.12*10^4$ PfSPZ are shown for early 28-day regimen subgroups B1 (only treated with chloroquine, CQ. n=4) and B2 (CQ + Zithromax Uno (AZ). n=5). Blue line represents median log_{10} parasites per milliliter of blood detected by quantitative PCR while grey patterns define the standard deviation of the data. The area under the curve (AUC) bar charts in b compare individual parasitemia detected in the 5–12-day interval after first immunization for both B1 and B2 subgroups.

Supplementary Figure 9

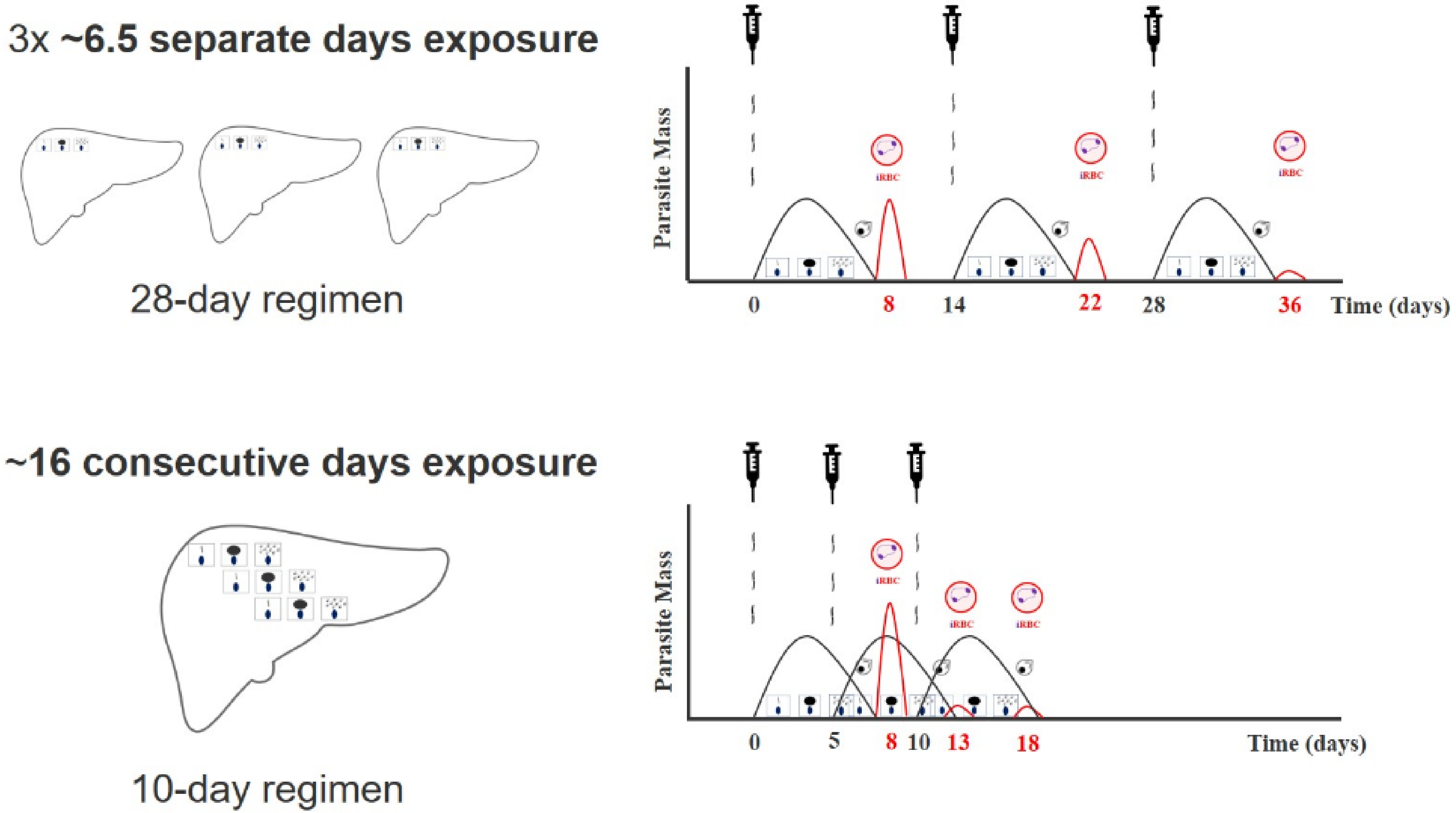

Supplementary Fig. 9. **Clinical trial design overview**. In short, two condensed immunization regimens comprising either three separate times of six days under malaria antigens exposure (28-day, Up) or sixteen consecutive days of continuous exposure to malaria antigens (10-day, Down) assessed in the subsequent phase B of the PfSPZ-CVac clinical trial (NCT02115516) are represented by cartoons. All participants were randomized and inoculated three times with 500 microliters of either a solution of sodium chloride 0.9% (Placebo) or a solution containing $5.12*10^4$ PfSPZ *Plasmodium falciparum* sporozoites; PfSPZ-CVac (Vaccinated). Immunizations were performed under chloroquine (CQ) chemoprophylaxis. All participants received a CQ loading dose of 10 mg/kg two days before first immunization and an additional CQ tablet of 5 mg/kg weekly to maintain the therapeutic regimen against malaria blood stages. Axis at the bottom show the expected waves of infected red blood cells (iRBCs) regarding the prepatent liver phase cycle of 7-9 days over time. Scale proportions aimed to facilitate the understanding of both schedules.

Supplementary Table 1. Demographics of the study participants.

Trial stage	Regimen	Arm	Allocation	Gender (M/F)	Age (years)	Height (cm)	Weight (kg)	BMI (kg/m2)
	28-day (B1/B2)	CQ	Placebo	2/1	30.3 (25.5-34.5)	181.5 (180-183)	84.5 (74-95)	25.7 (22.10-29.32)
PfSPZCVac	28-day (B1)	CQ	PfSPZ	3/0	29.2 (27.6-31.9)	180.8 (165-193)	77.6 (61-109)	23.4 (21.39-29.26)
Optimization	28-day (B2)	AZ/CQ	PfSPZ	3/3	26.1 (20.2-37.6)	176.8 (164-185)	82.2 (71-108)	26.1 (22.84-32.25)
(Tüchmi002B)	10-day (B3)	CQ	Placebo	2/0	24.5 (21.87-26.6)	173.3 (173-174)	79 (78-84)	26.2 (25.06-27.74)
	10-day (B3)	CQ	PfSPZ	4/4	28.1 (19.5-33.01)	172.2 (157-187)	77.4 (58-95)	26 (23.10-30.35)

Supplementary Table 1. **Baseline demographic characteristics of the study participants**. Numbers representing continuous variables (Age, Height, Weight, and Body Mass Index; BMI) show the mean plus the minimum and maximum values (in brackets) per each study arm.

Supplementary Table 2. Adverse events list reported for the immunization phase.

System Organ Class	Preferred Term	Placebo (N=6)	Verum (N=19)
Blood and lymphatic system disorders	#Total	[2] 1 (16.7%)	[18] 10 (52.6%)
	Leucocytosis		[1] 1 (5.3%)
	Leukopenia		[2] 2 (10.5%)
	Lymphocytosis		[1] 1 (5.3%)
	Lymphopenia		[4] 3 (15.8%)
	Monocytopenia		[1] 1 (5.3%)
	Monocytosis	[2] 1 (16.7%)	[7] 7 (36.8%)
	Neutropenia		[1] 1 (5.3%)
	Thrombocytopenia		[1] 1 (5.3%)
Cardiac disorders	#Total		[3] 2 (10.5%)
	Atrial flutter		[1] 1 (5.3%)
	Tachycardia		[2] 2 (10.5%)
Ear and labyrinth disorders	#Total		[1] 1 (5.3%)
	Ear discomfort		[1] 1 (5.3%)
Febrile disorders	#Total	[1] 1 (16.7%)	[3] 3 (15.8%)
	Pyrexia	[1] 1 (16.7%)	[3] 3 (15.8%)
Gastrointestinal disorders	#Total	[2] 2 (33.3%)	[20] 7 (36.8%)
	Abdominal discomfort	[1] 1 (16.7%)	[1] 1 (5.3%)
	Abdominal pain		[1] 1 (5.3%)
	Diarrhoea	[1] 1 (16.7%)	[11] 4 (21.1%)
	Flatulence		[1] 1 (5.3%)
	Gastrointestinal haemorrhage		[1] 1 (5.3%)
	Haematochezia		[1] 1 (5.3%)
	Nausea		[3] 3 (15.8%)
	Vomiting		[1] 1 (5.3%)
General disorders and administration site conditions	#Total	[4] 2 (33.3%)	[36] 15 (78.9%)
	Chills		[7] 6 (31.6%)
	Fatigue	[3] 2 (33.3%)	[17] 12 (63.2%)
	Hyperhidrosis	[1] 1 (16.7%)	[11] 10 (52.6%)
	Pyrexia		[1] 1 (5.3%)
Injury, poisoning and procedural complications	#Total	[1] 1 (16.7%)	[2] 2 (10.5%)
	Injection site erythema	[1] 1 (16.7%)	[1] 1 (5.3%)
	Injection site pain		[1] 1 (5.3%)
Investigations	#Total	[1] 1 (16.7%)	[30] 9 (47.4%)
	Alanine aminotransferase increased		[7] 5 (26.3%)
	Aspartate aminotransferase increased		[5] 5 (26.3%)
	Basophil count decreased		[1] 1 (5.3%)
	Blood lactate dehydrogenase increased	[1] 1 (16.7%)	[7] 7 (36.8%)
	Eosinophil count decreased		[1] 1 (5.3%)
	Eosinophil count increased		[2] 2 (10.5%)
	Haematocrit decreased		[3] 3 (15.8%)
	Haemoglobin decreased		[1] 1 (5.3%)
	Occult blood positive		[1] 1 (5.3%)
	Red blood cell count decreased		[2] 1 (5.3%)
Metabolism and nutrition disorders	#Total		[2] 2 (10.5%)
	Hyperbilirubinaemia		[1] 1 (5.3%)
	Hyperglycaemia		[1] 1 (5.3%)
Musculoskeletal and connective tissue disorders	#Total	[2] 2 (33.3%)	[7] 6 (31.6%)
	Arthralgia	[1] 1 (16.7%)	[2] 2 (10.5%)
	Myalgia	[1] 1 (16.7%)	[5] 5 (26.3%)
Nervous system disorders	#Total	[1] 1 (16.7%)	[23] 15 (78.9%)
	Dizziness		[3] 2 (10.5%)
	Headache	[1] 1 (16.7%)	[19] 14 (73.7%)
	Paraesthesia		[1] 1 (5.3%)
Psychiatric disorders	#Total	[2] 1 (16.7%)	[1] 1 (5.3%)
	Confusional state	[1] 1 (16.7%)	
	Disturbance in attention	[1] 1 (16.7%)	
	Insomnia		[1] 1 (5.3%)
Respiratory, thoracic and mediastinal disorders	#Total	[2] 1 (16.7%)	
	Acute upper respiratory tract infection	[1] 1 (16.7%)	
	Oropharyngeal pain	[1] 1 (16.7%)	

Supplementary Table 2. Adverse events list reported for the immunization phase. Related grade 1 and 2 AEs are listed using the MedDRA terminology. The statistical test used Fisher's exact test, two-sided for all. Square brackets are meant for the number of events followed by the number of participants affected and the percentage of participants with events (in brackets).

Supplementary Table 3. Adverse events list reported for CHMI.

System Organ Class	Preferred Term	Placebo (N=5)	Verum (N=14)
Blood and lymphatic system disorders	#Total	[3] 1 (20%)	[11] 7 (50%)
	Leucocytosis		[2] 2 (14.3%)
	Leukopenia	[2] 1 (20%)	
	Lymphopenia	[1] 1 (20%)	[3] 3 (21.4%)
	Monocytosis		[2] 2 (14.3%)
	Thrombocytopenia		[4] 3 (21.4%)
Cardiac disorders	#Total	[3] 3 (60%)	[5] 3 (21.4%)
	Tachycardia	[3] 3 (60%)	[5] 3 (21.4%)
Febrile disorders	#Total	[8] 5 (100%)	[8] 5 (35.7%)
	Pyrexia	[8] 5 (100%)	[8] 5 (35.7%)
Gastrointestinal disorders	#Total	[5] 3 (60%)	[5] 3 (21.4%)
	Abdominal pain		[1] 1 (7.1%)
	Diarrhoea	[2] 2 (40%)	
	Nausea	[3] 3 (60%)	[3] 2 (14.3%)
	Vomiting		[1] 1 (7.1%)
General disorders and administration site conditions	#Total	[10] 5 (100%)	[19] 7 (50%)
	Chills	[3] 3 (60%)	[4] 4 (28.6%)
	Fatigue	[4] 4 (80%)	[8] 7 (50%)
	Hyperhidrosis	[3] 3 (60%)	[7] 6 (42.9%)
Investigations	#Total	[7] 3 (60%)	[19] 9 (64.3%)
	Alanine aminotransferase increased	[1] 1 (20%)	[2] 2 (14.3%)
	Aspartate aminotransferase increased	[1] 1 (20%)	[2] 2 (14.3%)
	Blood creatinine increased	[1] 1 (20%)	
	Blood lactate dehydrogenase increased		[4] 4 (28.6%)
	Blood pressure diatolic increased		[3] 3 (21.4%)
	Blood pressure systolic increased		[1] 1 (7.1%)
	Eosinophil count decreased	[1] 1 (20%)	
	Haematocrit decreased	[2] 2 (40%)	[3] 3 (21.4%)
	Haemoglobin decreased	[1] 1 (20%)	[1] 1 (7.1%)
	Neutrophil count increased		[2] 2 (14.3%)
	Red blood cell count decreased		[1] 1 (7.1%)
Musculoskeletal and connective tissue disorders	#Total	[3] 3 (60%)	[7] 6 (42.9%)
	Arthralgia		[1] 1 (7.1%)
	Myalgia	[3] 3 (60%)	[6] 6 (42.9%)
Nervous system disorders	#Total	[8] 5 (100%)	[13] 8 (57.1%)
	Dizziness	[2] 2 (40%)	[3] 3 (21.4%)
	Headache	[6] 5 (100%)	[10] 8 (57.1%)
Psychiatric disorders	#Total		[1] 1 (7.1%)
	Insomnia		[1] 1 (7.1%)
Renal and urinary disorders	#Total		[1] 1 (7.1%)
	Costovertebral angle tenderness		[1] 1 (7.1%)
Respiratory, thoracic and mediastinal disorders	#Total	[2] 1 (20%)	[2] 2 (14.3%)
	Oropharyngeal pain	[2] 1 (20%)	[2] 2 (14.3%)
Vascular disorders	#Total		[1] 1 (7.1%)
	Haematuria		[1] 1 (7.1%)

Supplementary Table 3. Adverse events list reported for CHMI. Related grade 1 and 2 AEs are listed using the MedDRA terminology. The statistical test used Fisher’s exact test, two-sided for all. Square brackets are meant for the number of events followed by the number of participants affected and the percentage of participants with events (in brackets).
