# Supplementary material for: Efficacy, T cell activation and antibody responses in accelerated Plasmodium falciparum sporozoite chemoprophylaxis vaccine regimens
Source: NPJ Vaccines. 2022 May 31;7:59. doi: 10.1038/s41541-022-00473-1 (PMC9156686; doi:10.1038/s41541-022-00473-1)
Supplement: Supplementary file 2 — REPORTING SUMMARY [file 41541_2022_473_MOESM2_ESM.pdf]

nature portfolio

Corresponding author(s): Benjamin Mordmüller

Last updated by author(s): Dec 14, 2021

Reporting Summary

Nature Portfolio wishes to improve the reproducibility of the work that we publish. This form provides structure for consistency and transparency in reporting. For further information on Nature Portfolio policies, see our Editorial Policies and the Editorial Policy Checklist.

Statistics

For all statistical analyses, confirm that the following items are present in the figure legend, table legend, main text, or Methods section.

n/a	Confirmed	
☐	☒	The exact sample size (*n*) for each experimental group/condition, given as a discrete number and unit of measurement
☐	☒	A statement on whether measurements were taken from distinct samples or whether the same sample was measured repeatedly
☐	☒	The statistical test(s) used AND whether they are one- or two-sided<br>*Only common tests should be described solely by name; describe more complex techniques in the Methods section.*
☒	☐	A description of all covariates tested
☐	☒	A description of any assumptions or corrections, such as tests of normality and adjustment for multiple comparisons
☐	☒	A full description of the statistical parameters including central tendency (e.g. means) or other basic estimates (e.g. regression coefficient) AND variation (e.g. standard deviation) or associated estimates of uncertainty (e.g. confidence intervals)
☐	☒	For null hypothesis testing, the test statistic (e.g. *F*, *t*, *r*) with confidence intervals, effect sizes, degrees of freedom and *P* value noted<br>*Give P values as exact values whenever suitable.*
☒	☐	For Bayesian analysis, information on the choice of priors and Markov chain Monte Carlo settings
☒	☐	For hierarchical and complex designs, identification of the appropriate level for tests and full reporting of outcomes
☒	☐	Estimates of effect sizes (e.g. Cohen's *d*, Pearson's *r*), indicating how they were calculated

Our web collection on statistics for biologists contains articles on many of the points above.

Software and code

Policy information about availability of computer code

Data collection	Open clinica was used for data collection.
Data analysis	R 3.4.3<br>GraphPad Prism 9

For manuscripts utilizing custom algorithms or software that are central to the research but not yet described in published literature, software must be made available to editors and reviewers. We strongly encourage code deposition in a community repository (e.g. GitHub). See the Nature Portfolio guidelines for submitting code & software for further information.

Data

Policy information about availability of data

All manuscripts must include a data availability statement. This statement should provide the following information, where applicable:

- Accession codes, unique identifiers, or web links for publicly available datasets
- A description of any restrictions on data availability
- For clinical datasets or third party data, please ensure that the statement adheres to our policy

All data supporting the findings of this study are available upon reasonable request from the corresponding author.

Field-specific reporting

Please select the one below that is the best fit for your research. If you are not sure, read the appropriate sections before making your selection.

☒ Life sciences ☐ Behavioural & social sciences ☐ Ecological, evolutionary & environmental sciences

For a reference copy of the document with all sections, see nature.com/documents/nr-reporting-summary-flat.pdf

Life sciences study design

All studies must disclose on these points even when the disclosure is negative.

Sample size	The sample size was calculated using the function nBinomial of package gsDesign of R version49 which estimates the sample size required to detect a difference between two rates.
Data exclusions	No data were excluded.
Replication	*Describe the measures taken to verify the reproducibility of the experimental findings. If all attempts at replication were successful, confirm this OR if there are any findings that were not replicated or cannot be reproduced, note this and describe why.*
Randomization	According to study protocol, treatment allocation was random and blinded with a ratio of 2:1 (PfSPZ: placebo). A dedicated member of the formulation team, who was not involved in volunteer management or diagnostic activities, was responsible for keeping the randomization envelopes and dosing schedule while a third party outside the study team and sponsor, generated and distributed the randomization list.
Blinding	Twenty-two healthy malaria-naïve young adult volunteers out of 46 assessed for eligibility were chosen following a randomized double-blinded placebo-controlled immunization trial to explore and compare immunogenicity. The PfSPZ formulation team was unblinded and thus was not involved in clinical or diagnostic activities. The IMP and the Placebo syringes were blinded by the formulation team.

Reporting for specific materials, systems and methods

We require information from authors about some types of materials, experimental systems and methods used in many studies. Here, indicate whether each material, system or method listed is relevant to your study. If you are not sure if a list item applies to your research, read the appropriate section before selecting a response.

Materials & experimental systems

n/a	Involved in the study	
☐	☒	Antibodies
☒	☐	Eukaryotic cell lines
☒	☐	Palaeontology and archaeology
☒	☐	Animals and other organisms
☐	☒	Human research participants
☐	☐	Clinical data
☐	☐	Dual use research of concern

Methods

n/a	Involved in the study	
☐	☐	ChIP-seq
☐	☐	Flow cytometry
☐	☐	MRI-based neuroimaging

Antibodies

Antibodies used	Miltenyi:<br>anti-CD40 (#Cat 130-094-133)<br>anti-CD28 (#Cat 130-093-375)<br>anti-CD40L-Biotin #Cat 130-092-658<br>anti-CD8-PerCP (#Cat 130-008-057), anti-CD14-PerCP (#Cat 130-098-072), anti-CD20-PerCP (#Cat 130-098-097), anti-CD4-VioBlue (#Cat 130-099-683), anti-CD45RO-PE-Vio 770 (#Cat 130-099-692)<br><br>antiCD8-VioGreen (#Cat 130-096-902), anti-CD14-VioGreen (#Cat 130-096-875), anti-CD20-VioGreen (#Cat 130-096-094), anti-CD4-APC-Vio770 (#Cat 130-100-457), anti-CD45RO-FITC (#Cat 130-095-462), anti-CCR7-PE (#Cat 130-099-361) anti-CD40L-VioBlue (#Cat 130-096-217), anti-TNF-α-PE-Vio 770 (6 µl) (#Cat 130-096-755)<br>anti-CCR7-FITC (UCHL-1; Becton Dickinson Biosciences)<br>anti-IFN-γ-PerCP 5.5 (0.6 µl) (Biolegend; #Cat 502526)
Validation	All antibodies are commercially available and verified by the stated companies.

Human research participants

Policy information about studies involving human research participants

Population characteristics	Demographics are summarized in Supplementary Table 1.
Recruitment	We recruited healthy, non-pregnant, malaria naive volunteers aged between 18 and 45 years using the university email list.
Ethics oversight	Approval was obtained from the Ethics Committee of the Eberhard Karls University and University Clinics (EudraCT number 2013-003900-38, National Clinical Trial number (NCT): NCT02115516). The study was performed in accordance with Good Clinical Practice/ International Conference on Harmonization guidelines.

Note that full information on the approval of the study protocol must also be provided in the manuscript.

Clinical data

Policy information about clinical studies

All manuscripts should comply with the ICMJE guidelines for publication of clinical research and a completed CONSORT checklist must be included with all submissions.

Clinical trial registration	National Clinical Trial number (NCT): NCT02115516
Study protocol	The study protocol is attached to the manuscript.
Data collection	This single center, randomized, placebo-controlled, double-blinded phase I/II study was conducted from April 2015 to December 2015 at the Institute of Tropical Medicine in Tübingen, Germany.
Outcomes	Primary efficacy endpoint was assigned to the proportion of volunteers parasitemic within 21 days after CHMI while secondary efficacy endpoint considered the time to detect parasitemia (pre-patent period). Primary safety endpoint was set as the occurrence of related Grade 3 adverse events (AEs) from first chemoprophylactic dose uptake (I-2) and PfSPZ challenge administration (I) until the end of the study. Secondary safety point focused on the appearance of any related AE from time of first administration of an immunizing regimen (PfSPZ-CVac) until the end of the study.

Dual use research of concern

Policy information about dual use research of concern

Hazards

Could the accidental, deliberate or reckless misuse of agents or technologies generated in the work, or the application of information presented in the manuscript, pose a threat to:

No	Yes	
☒	☐	Public health
☒	☐	National security
☒	☐	Crops and/or livestock
☒	☐	Ecosystems
☒	☐	Any other significant area

Experiments of concern

Does the work involve any of these experiments of concern:

No	Yes	
☒	☐	Demonstrate how to render a vaccine ineffective
☒	☐	Confer resistance to therapeutically useful antibiotics or antiviral agents
☒	☐	Enhance the virulence of a pathogen or render a nonpathogen virulent
☒	☐	Increase transmissibility of a pathogen
☒	☐	Alter the host range of a pathogen
☒	☐	Enable evasion of diagnostic/detection modalities
☒	☐	Enable the weaponization of a biological agent or toxin
☒	☐	Any other potentially harmful combination of experiments and agents

ChIP-seq

Data deposition

☐ Confirm that both raw and final processed data have been deposited in a public database such as GEO.

☐ Confirm that you have deposited or provided access to graph files (e.g. BED files) for the called peaks.

Data access links<br>*May remain private before publication.*	*For "Initial submission" or "Revised version" documents, provide reviewer access links. For your "Final submission" document, provide a link to the deposited data.*
Files in database submission	*Provide a list of all files available in the database submission.*
Genome browser session<br>(e.g. UCSC)	*Provide a link to an anonymized genome browser session for "Initial submission" and "Revised version" documents only, to enable peer review. Write "no longer applicable" for "Final submission" documents.*

Methodology

Replicates	*Describe the experimental replicates, specifying number, type and replicate agreement.*
Sequencing depth	*Describe the sequencing depth for each experiment, providing the total number of reads, uniquely mapped reads, length of reads and whether they were paired- or single-end.*
Antibodies	*Describe the antibodies used for the ChIP-seq experiments; as applicable, provide supplier name, catalog number, clone name, and lot number.*
Peak calling parameters	*Specify the command line program and parameters used for read mapping and peak calling, including the ChIP, control and index files used.*
Data quality	*Describe the methods used to ensure data quality in full detail, including how many peaks are at FDR 5% and above 5-fold enrichment.*
Software	*Describe the software used to collect and analyze the ChIP-seq data. For custom code that has been deposited into a community repository, provide accession details.*

Flow Cytometry

Plots

Confirm that:

☒ The axis labels state the marker and fluorochrome used (e.g. CD4-FITC).

☒ The axis scales are clearly visible. Include numbers along axes only for bottom left plot of group (a 'group' is an analysis of identical markers).

☒ All plots are contour plots with outliers or pseudocolor plots.

☒ A numerical value for number of cells or percentage (with statistics) is provided.

Methodology

Sample preparation

Peripheral blood mononuclear cells (PBMCs) were obtained by ficoll-paque plus density gradient centrifugation (GE Healthcare Life Science, ref# 17-1440-02) from the heparinized whole blood. All assays were performed using fresh PBMC samples.
In brief, PBMCs from each participant were placed in RPMI 1640 medium (Sigma-Aldrich), supplemented with 5% (v/v) AB serum (Lonza) and 2 mM L-glutamine (PAA Laboratories) for 18h at 37°Cat 5% CO2. Hence, in order to enrich the Ag-specific T cells plate-wells containing 1*107 PBMCs were stimulated for 5 h with the following stimulants: a) negative control; 500 µl of thawed uninfected red blood cells (RBC) from a culture collection aliquoted and stored at -80°C, b) 500 µl of a thawed aliquot containing infected red blood cells (iRBCs, Pf3D7 laboratory strain; 90% schizonts by microscopy) and c) 5 µg/ml of Staphylococcus endotoxin B (SEB, Sigma-Aldrich; ref# S4881) as a positive control. All samples reached a final volume of 1500 µl/well.
In addition to the stimulants cited above, all samples were incubated in the presence of 1 µg/ml anti-CD40 (#Cat 130-094-133) and 1 µg/ml anti-CD28 (#Cat 130-093-375) functional grade pure antibody (Ab) (both Miltenyi Biotec). Brefeldin A (Sigma; #Cat B7151) was employed at 1 µg/ml for 2h to stop cytokine release. As a negative control, non-autologous uninfected RBCs (uRBCs; type O+) obtained from the blood donation center (Zentrum für Klinische Transfusionmedizin Tübingen gemeinnützige GmbH) were used for each participant.
After incubation, a small fraction of 30 µl from the original sample (ORI) was taken to assess the original phenotyping before magnetic separation by direct staining with 65 µl of the original fraction staining mix. The leftover sample was centrifuge at 300 xg for 5 minutes. The pelleted cells were labeled with 10µl of anti-CD40L-Biotin (Miltenyi Biotec; #Cat 130-092-658) for 10 minutes at 4°C. Samples were washed two times with PEB buffer (1xPBS, 2mM EDTA and 0.5% BSA) before being loaded into the magnetic columns to perform the anti-biotin magnetic sorting separation (MACS) (Miltenyi Biotec; #Cat 130-042-201).
Once the columns had retained the coated cells, 60 µl of the staining mix for the surface staining was added to each column. Following, 1xPBS (Gibco; Life technologies) was used two times before eluting the sample with 500 µl of PEB. The eluted cells

	were collected in 1.5 ml tubes (Eppendorf) to be fixed with 200 µl of Fixable Live/Dead (Inside stain kit; Miltenyi Biotec).<br>A second round of magnetic separation was performed over the fixed suspension using a new set of columns. Previously, a rising step with 500 µl of PEB was done to prepare the new columns.<br>Once the fixed positive fraction was retained again, the columns were washed out with 500 µl of PEB before adding 200 µl of Inside Perm buffer (Inside stain kit; Miltenyi Biotec; #Cat 130-090-477) into the column. Then, 60 µl of the intracellular staining cocktail was added to every column. After 15 minutes of incubation at room temperature, columns were washed out with 200 µl of Inside Perm buffer. Eluted cells with 1000 µl of PEB buffer were collected into 1.5ml Eppendorf tubes to be centrifuged at 300g for 5 minutes. Pellet was resuspended in 200 µl of PEB buffer to Flow cytometry analysis. If not mentioned otherwise, the monoclonal antibodies were purchased from Miltenyi Biotech.
Instrument	BD FACS Canto II
Software	FlowJo V10 was used to analyze flow cytometry data. Both statistical analysis and figures were generated using GraphPad Prism 8.1.2 (GraphPad Software, San Diego, CA) and FACSDiva software (BD Biosciences).
Cell population abundance	The remaining sample of each well is magnetically isolated to enrich the CD40L+ population stimulated with uRBC, iRBC or SEB (step 2). The resulting positive samples (PS) are analyzed by flow cytometry for the intracellular staining of TNF-α and IFN-γ. Moreover, each subpopulation of mono or polyfunctional T cells was classified into effector memory T cells (TEM), central memory T cells (TCM) and naïve T cells (TN) using the antibodies anti-CD45RO and anti-CCR7 (Supplementary Fig. 3 and Supplementary Fig. 4).<br>In step 3, the net percentage of iRBC-specific CD40L+CD4+ T cells is calculated according to the formula (Supplementary Fig. 6):<br>(iRBC stimulated TEM events-uRBC stimulated TEM events)/(fraction of iRBC stimulated Th cells*10^7 Total stimulated PBMCs) *100%
Gating strategy	The gating strategy is shown in detail in supplementary figure 3, 4 and 5.

☒ Tick this box to confirm that a figure exemplifying the gating strategy is provided in the Supplementary Information.

Magnetic resonance imaging

Experimental design

Design type	*Indicate task or resting state; event-related or block design.*
Design specifications	*Specify the number of blocks, trials or experimental units per session and/or subject, and specify the length of each trial or block (if trials are blocked) and interval between trials.*
Behavioral performance measures	*State number and/or type of variables recorded (e.g. correct button press, response time) and what statistics were used to establish that the subjects were performing the task as expected (e.g. mean, range, and/or standard deviation across subjects).*

Acquisition

Imaging type(s)	*Specify: functional, structural, diffusion, perfusion.*
Field strength	*Specify in Tesla*
Sequence & imaging parameters	*Specify the pulse sequence type (gradient echo, spin echo, etc.), imaging type (EPI, spiral, etc.), field of view, matrix size, slice thickness, orientation and TE/TR/flip angle.*
Area of acquisition	*State whether a whole brain scan was used OR define the area of acquisition, describing how the region was determined.*

Diffusion MRI ☐ Used ☐ Not used

Preprocessing

Preprocessing software	*Provide detail on software version and revision number and on specific parameters (model/functions, brain extraction, segmentation, smoothing kernel size, etc.).*
Normalization	*If data were normalized/standardized, describe the approach(es): specify linear or non-linear and define image types used for transformation OR indicate that data were not normalized and explain rationale for lack of normalization.*
Normalization template	*Describe the template used for normalization/transformation, specifying subject space or group standardized space (e.g. original Talairach, MNI305, ICBM152) OR indicate that the data were not normalized.*
Noise and artifact removal	*Describe your procedure(s) for artifact and structured noise removal, specifying motion parameters, tissue signals and physiological signals (heart rate, respiration).*
Volume censoring	*Define your software and/or method and criteria for volume censoring, and state the extent of such censoring.*

Statistical modeling & inference

Model type and settings

Specify type (mass univariate, multivariate, RSA, predictive, etc.) and describe essential details of the model at the first and second levels (e.g. fixed, random or mixed effects; drift or auto-correlation).

Effect(s) tested

Define precise effect in terms of the task or stimulus conditions instead of psychological concepts and indicate whether ANOVA or factorial designs were used.

Specify type of analysis: ☐ Whole brain ☐ ROI-based ☐ Both

Statistic type for inference
(See Eklund et al. 2016)

Specify voxel-wise or cluster-wise and report all relevant parameters for cluster-wise methods.

Correction

Describe the type of correction and how it is obtained for multiple comparisons (e.g. FWE, FDR, permutation or Monte Carlo).

Models & analysis

n/a	Involved in the study
☐	☐ Functional and/or effective connectivity
☐	☐ Graph analysis
☐	☐ Multivariate modeling or predictive analysis

Functional and/or effective connectivity

Report the measures of dependence used and the model details (e.g. Pearson correlation, partial correlation, mutual information).

Graph analysis

Report the dependent variable and connectivity measure, specifying weighted graph or binarized graph, subject- or group-level, and the global and/or node summaries used (e.g. clustering coefficient, efficiency, etc.).

Multivariate modeling and predictive analysis

Specify independent variables, features extraction and dimension reduction, model, training and evaluation metrics.
